# *SlJAZ10* and *SlJAZ11* mediate dark-induced leaf senescence and regeneration

**DOI:** 10.1371/journal.pgen.1010285

**Published:** 2022-07-13

**Authors:** Boyan Tang, Tingting Tan, Yating Chen, Zongli Hu, Qiaoli Xie, Xiaohui Yu, Guoping Chen

**Affiliations:** 1 Key Laboratory of Bioengineering Science and Technology, Chongqing University, Ministry of Education, Chongqing, China; 2 Bioengineering College, Campus B, Chongqing University, Chongqing, People’s Republic of China; 3 Hainan Key Laboratory for Sustainable Utilization of Tropical Bioresources, College of Tropical Crops, Hainan University, Haikou, People’s Republic of China; Peking University, CHINA

## Abstract

During evolutionary adaptation, the mechanisms for self-regulation are established between the normal growth and development of plants and environmental stress. The phytohormone jasmonate (JA) is a key tie of plant defence and development, and JASMONATE-ZIM DOMAIN (JAZ) repressor proteins are key components in JA signalling pathways. Here, we show that JAZ expression was affected by leaf senescence from the transcriptomic data. Further investigation revealed that *SlJAZ10* and *SlJAZ11* positively regulate leaf senescence and that *SlJAZ11* can also promote plant regeneration. Moreover, we reveal that the SlJAV1-SlWRKY51 (JW) complex could suppress JA biosynthesis under normal growth conditions. Immediately after injury, SlJAZ10 and SlJAZ11 can regulate the activity of the JW complex through the effects of electrical signals and Ca^2+^ waves, which in turn affect JA biosynthesis, causing a difference in the regeneration phenotype between *SlJAZ10-OE* and *SlJAZ11-OE* transgenic plants. In addition, SlRbcs-3B could maintain the protein stability of SlJAZ11 to protect it from degradation. Together, SlJAZ10 and SlJAZ11 not only act as repressors of JA signalling to leaf senescence, but also regulate plant regeneration through coordinated electrical signals, Ca^2+^ waves, hormones and transcriptional regulation. Our study provides critical insights into the mechanisms by which SlJAZ11 can induce regeneration.

## Author summary

In plants, senescence is the final stage of development, but regeneration can help them beyond the stage. Plants regeneration is essential for propagation, and in cultivated crops to maintain excellent traits as close as possible. JA signaling can sense environmental signals and integrate various regulatory mechanisms to ensure plants regeneration occurs under optimal conditions. In this work, the JAZ-JAV1-WRKY51 complexes with reported was further optimized, the function of SlJAZ10 and SlJAZ11 was identified to promote inhibitory activity of SlJAV1-SlWRKY51 complex which negatively regulated JA biosynthesis by direct binding of the W-box of the *SlAOC* promoter. The results of further investigation suggest that the differences in regulation of electrical signals, Ca^2+^ waves, hormones and transcriptional regulation are responsible for the regeneration between SlJAZ10 and SlJAZ11. In addition, we have found that SlRbcs-3B could maintain the protein stability of SlJAZ11 to protect it from degradation. In summary, despite both SlJAZ10 and SlJAZ11 can function as senescence, only SlJAZ11 has an important promoting function for regeneration.

## Introduction

Leaf ageing, eventually senescence or structural damage of leaf structures, is a crucial link of later stages in plant growth and development [1]. Leaf senescence is not simply a termination phase for leaf life, but also is more of a programmed plan for nutrient relocation within the plant [2–4]. A series of complex changes in physiology, biochemical metabolism, hormone levels, and gene regulation are accompany leaf senescence [5]. Generally, leaf senescence has been divided into leaf senescence-developmental (natural) leaf senescence and stress-induced leaf senescence. Natural leaf senescence is an age-dependent process and preludes to leaf death [6], while stress-induced leaf senescence is induced by an external stimulus [7]. Stress-induced leaf senescence may induce premature senescence and even affect plant growth, which can lead to a decrease in plant yield and quality [8, 9]. Moreover, prolonged darkness is a well-known powerful external stimulus capable of accelerating leaf senescence [10].

Plant regeneration is a unique phenomenon credited to cellular totipotency. Regeneration capacity is associated with cellular senescence and ageing, which are frequently associated with wound signals [11]. Plant regeneration involves the stimulation of exogenous plant hormone signalling, the division of quiescent cells, and formation of meristems or organ primordia in vitro [12]. The molecular crossroads between stress responses and regeneration have begun to be studied in recent years. During regeneration after injury, jasmonic acid immediately accumulates and activates ERF109 (ETHYLENE RESPONSE FACTOR109). Immediately afterwards, ERF109 upregulates ASA1 (ANTHRANILATE SYNTHASE α1) which can mediate lateral root formation. Hours later, ERF109 initiates a regulatory feedback loop to repress JA signalling pathways by directly interacting with JASMONATEZIM-DOMAIN (JAZ) proteins [13–16].

Jasmonate has been well recognized as a plant-defending hormone involved in leaf senescence, regeneration, abiotic stress response, wounding and pathogen responses [17–20]. Ros, calcium, and electric signals are the key mediators of rapid signals in response to ageing [17]. JA has been demonstrated to be a vital signal that triggers plant responses against abiotic stress and pathogen infection [21]. JA not only can interact with auxin, gibberellin and ethylene to regulate leaf senescence [22], but also serves as a potent regulatory lipid in response to stress [23]. By adjusting the relative JA contents in wounds and in undamaged distal tissues, the plant-defence systems are activated against various stresses [24]. The latest literature has revealed that wounding and JA are the first events triggering regeneration [24–27].

The biologically active form of JA, Jasmonoyl-isoleucine (JA-Ile) is an essential step for JA signalling responses. In plants, CORONATINE INSENSITIVE 1 (COI1), JASMONATE ZIM-DOMAIN proteins (JAZs) and JAZ-interacting transcription factors have proven to be crucial for the perception of JA signals and the subsequent signal transduction pathway steps [28]. Upon JA-Ile perception, JAZ proteins are specifically bound by COI1, leading to polyubiquitylation and subsequent degradation of JAZ by the 26S proteasome [21]. Degradation of JAZ leads to the expression of jasmonate-responsive genes and derepresses transcription factors such as MYC2 [2, 29, 30]. As repressors for JA signalling, JASMONAT-ZIM-domain (JAZ) proteins play critical roles in mediating various aspects of the JA response [31]. In addition to the repressor and coreceptor functions of JA signalling, accumulating evidence has shown that JAZ proteins play an integral role in regulating the desensitization of the JA-related pathway, which is essential to preventing uncontrolled JA responses [32].

In tomato, the JAZ family of repressors is composed of an atypical repressor (*SlJAZ13*) and 12 canonical members (*SlJAZ1* to *SlJAZ12*). Individual JAZ genes can exert diverse functions in different plant tissues. As a model plant, *Arabidopsis thaliana* was primarily approved for the JA signalling pathway. Recently, major discoveries about the JA signalling pathway have been made in the model plant (Arabidopsis thaliana). Overall, JAZs can play critical roles in the regulation of plant growth and development, and also are essential for controlling pests and diseases [32–35]. However, most of the latest JA-related research has focused on regeneration [19, 36–38]. To date, it has been clarified that JA can induce stem cell activation and regeneration and activate growth after wounding. Nevertheless, the function of JAZ proteins in plant regeneration are needed to be further investigated.

Here, we report that *SlJAZ10* and *SlJAZ11* are involved in leaf senescence and regeneration. Next, we verified that SlJAZ10-SlJAV1-SlWRKY51 (JJW) and SlJAZ11-SlJAV1-SlWRKY51 (JJW) complexes bind and regulate JA biosynthesis genes. Genetic analysis of overexpression lines demonstrated that SlJAZ10 and SlJAZ11 induce self-propagating electrical activity, calcium influx and hormone aggregation after wounding. We also uncovered a new regulatory step in which SlRbcs-3B (ribulose bisphosphate carboxylase) can stabilize the SlJAZ11 protein and protect it from degradation of JAZ by the 26S proteasome.

## Materials and methods

### Plant growth conditions and materials

Tomato (*Solanum lycopersicum* Mill. cv. Ailsa Craig) was selected as wild-type (WT). Tomato seeds were placed on moistened filter paper for 72 h for germination. Tomato plants were grown in a growth chamber at controlled temperature (28°C during the day, 22°C at night) under a 16 h light photoperiod. *Nicotiana benthamiana* plants used for bimolecular fluorescence complementation (BiFC) and dual-luciferase (LUC) assay were grown in the growth chamber with temperature at 26°C and 16 h-8 h, light-dark cycles. All samples were immediately transferred to liquid nitrogen and stored at -80°C until required.

### Vector construction and plant transformation

To overexpress *SlJAZ10* and *SlJAZ11*, we amplified the full-length coding sequences (CDSs) of *SlJAZ10* and *SlJAZ11* with *Solanum lycopersicum* Max Super-Fidelity DNA and cloned them into pBI121 vector carrying the cauliflower mosaic virus (CaMV) 35S promoter. To generate CRISPR/Cas9-*SlJAZ10* and CRISPR/Cas9-*SlJAZ11* construct, we inserted two target sites of *SlJAZ10* and *SlJAZ11* (http://skl.scau.edu.cn/targetdesign) into the pTX vector with Clone Express II One Step Cloning Kit. By *Agrobacterium* (Agrobacterium tumefaciens)-mediated transformation, all constructs were introduced into tomato cv *Ailsa Craig*. Homozygous transgenic plants were used for phenotypic and molecular characterization. The primers used for gene were listed in S1 Table; a standard curve was performed for each pair of specific primers.

### Dark treatment for leaf senescence and biochemical assays

Tomato plants were grown in soil for 8 weeks, and the sixth or seventh leaf was detached for dark treatment. The detached leaves were incubated in Petri dishes with two layers of wet filter paper in a dark environment.

TUNEL staining was performed using the DeadEnd Fluorometric TUNEL System. Chlorophyll was extracted in 80% acetone and absorbance measured at 663 and 646 nm for calculation of total chlorophyll content per gram fresh weight. Measurement of malondialdehyde (MDA) was performed with a spectrophotometric assay. Reactive oxygen species (ROS) generation in MRSA was tested with a ROS assay kit. Glutathione (GSH) was measured with the micro reduced glutathione (GSH) assay kit.

### RNA isolation and real-time PCR analysis

Total RNA was extracted from various samples with RNAiso plus. First-strand cDNA which was synthesized with a kit. Quantitative reverse-transcription–PCR (qRT–PCR) was performed by Real-Time System. A no-template control was also included in each gene study. All qRT–PCR was performed with three replicates. The primers used for gene were listed in S2 Table; a standard curve was performed for each pair of specific primers.

### RNA-sequencing and data analysis

Differentially expressed genes were identified with DESeq2 with a filter threshold of adjusted q-value <0.05 and |log2FoldChange| > 1. Cluster Profiler (http://www.bioconductor.org/packages/release/bioc/html/clusterProfiler.html) in R package [39] was employed to perform GO and KEGG (http://www.genome.jp/kegg/) enrichment analysis [40]. The GO and KEGG enrichment analysis were calculated using hypergeometric distribution with a Q value cutoff of 0.05. Q values obtained by Fisher’s exact test were adjusted with FDR for multiple comparisons [41].

### Hormone determination analysis

Hormone determination analysis was carried out by high performance liquid chromatography (HPLC). Unsupervised PCA (principal component analysis) was performed by statistics function prcomp within R (www.r-project.org). The data was unit variance scaled before unsupervised PCA. The HCA (hierarchical cluster analysis) results of samples and metabolites were presented as heatmaps with dendrograms, while pearson correlation coefficients (PCC) between samples were calculated by the cor function in R and presented as only heatmaps. Both HCA and PCC were carried out by R package pheatmap. For HCA, normalized signal intensities of metabolites (unit variance scaling) were visualized as a color spectrum.

### Ibuprofen (IBU) and 2,3,5-triiodobenzoic acid (TIBA) treatment

For the IBU and TIBA treatment, tomato seeds were firstly germinated in a shaker with temperature set at 28°C and shaker speed 250 r/min for 72 h, then were transferred to ½MS agar medium with or without 100 μM IUB and TIBA in tissue culture flasks for 10 d.

### Subcellular localization assay

For subcellular localization, we used *Agrobacterium tumefaciens* strain GV3101 carrying 35S-SlJAZ10-GFP and 35S-SlJAZ11-GFP together with strain p19 (each strain, OD600 = 1.0, 1:1 ratio). At 3d after agroinfiltration, we detected the GFP signals with a confocal microscope. The primers used for gene were listed in S3 Table; a standard curve was performed for each pair of specific primers.

### Yeast two-hybrid (Y2H) and Biomolecular Fluorescent Complementation (BiFC) assay

For the Y2H, the Matchmaker GAL4-based two-hybrid system was utilized. Yeast strain Y2H was co-transformed with the specific bait and prey constructs and plated on the SD/Leu/-Trp/-His/-Ade drop-out medium and screened with β-galactosidase.

*N*. *benthamiana* leaves were used for BiFC. At 3 days after infiltration, YFP fluorescence was observed with a TCS SP5 confocal spectral microscope imaging system. The primers used for gene were listed in S4 Table; a standard curve was performed for each pair of specific primers.

### Pull-down assay

The CDS of *SlJAZ10* and *SlJAZ11* were cloned into pET-28a, and *SlRbcs-3B* into pGEX-4T. These resulted constructs were transformed into *Escherichia coli* BL21 (DE3) to produce recombinant proteins. For pull-down assay, GST-SlJAZ10, GST-SlJAZ11 or GST was incubated with GST Bind Resin at 4°C for 2 h, and then 0.5 mg of purified recombinant protein with His tag was added. The incubation continued for another 6 h, and the beads were washed with pull-down buffer for five times. The bounded proteins were finally eluted, and the pulled down proteins were analyzed by western-blot with the anti-His antibody and anti-GST antibody. The primers used for gene were listed in S5 Tbale.

### Dual-luciferase assay, transcriptional activity and Yeast one hybrid (Y1H)

LUC activity was measured by a dual luciferase assay kit. The *N*. *benthamiana* leaves were mixed in the PBS 72 h after infiltration. The ratio of LUC to REN was calculated indicate the final transcriptional activity.

Based on the GAL4/UAS-based system, SlJAZ10 and SlJAZ11 transcriptional activity was examined by a dual-LUC reporter assay system.

Y1H experiments were peformed according to the Matchmaker Gold Y1H System protocol. The bait plasmids were transformed into the Y1H Gold strain according to the manufacturer’s instructions. Aureobasidin A (AbA) was used to screen the minimal inhibitory concentration for the bait strains. The prey plasmid was transformed into a bait yeast strain to determine the DNA-protein interaction by screening them on SD medium with AbA and without leucine. The primers used for gene were listed in S6 Table; a standard curve was performed for each pair of specific primers.

### Surface potential recordings

For surface potential recordings, silver electrodes of 0.5 mm diameter were chloridized with HCl (0.1 M), stored at room temperature and rechloridized after several uses. Experiments were conducted in a controlled environment. High impedance amplifier was simultaneously used to record the surface potential at wound positions. The electrode–leaf interface was a drop (10 ml) of 10 mM KCl in 0.5% (w/v) agar placed so that the silver electrode did not contact and damage the cuticle. The ground electrode was placed in the soil. Raw data of surface potentials was acquired with a NI USB 6259 interface, using custom-built Labview-based software.

### Detection of intracellular free Ca^2+^ distribution using Fluo-3/AM staining

The 1.0 mM CaCl_2_-treated leaves were probed with Fluo-3/AM to detect the distribution of intracellular free Ca^2+^ ([Ca^2+^]_cyt_) in the cells. The levels of intracellular free Ca^2+^ in leaves were visualized with a live with excitation wavelength of 488 nm and emission wavelength of 525 nm, and the Ca^2+^ fluorescence intensity was quantified with Image Browser software.

### Vitro degradation assays

For in vitro degradation assays, total protein was extracted from the *N*. *benthamiana* leaves 3 days after injection with native extraction buffer (50 mM Tris-MES, pH8.0, 0.5 M sucrose, 1 mM MgCl_2_, 10 mM EDTA, 5 mM DTT, 1 mM PMSF, and 1*C-complete protease inhibitor). The extracted proteins were then blotted onto nitrocellulose membranes, blocked with nonfat dry milk, and incubated with anti-ACTIN antibody. Protein gel blot pictures were scanned, and the intensity of the images was quantified by ImageJ. The primers used for gene were listed in S7 Table; a standard curve was performed for each pair of specific primers.

## Results

### JAZ genes respond to the senescence of detached leaves in tomato

**Fig 1:**
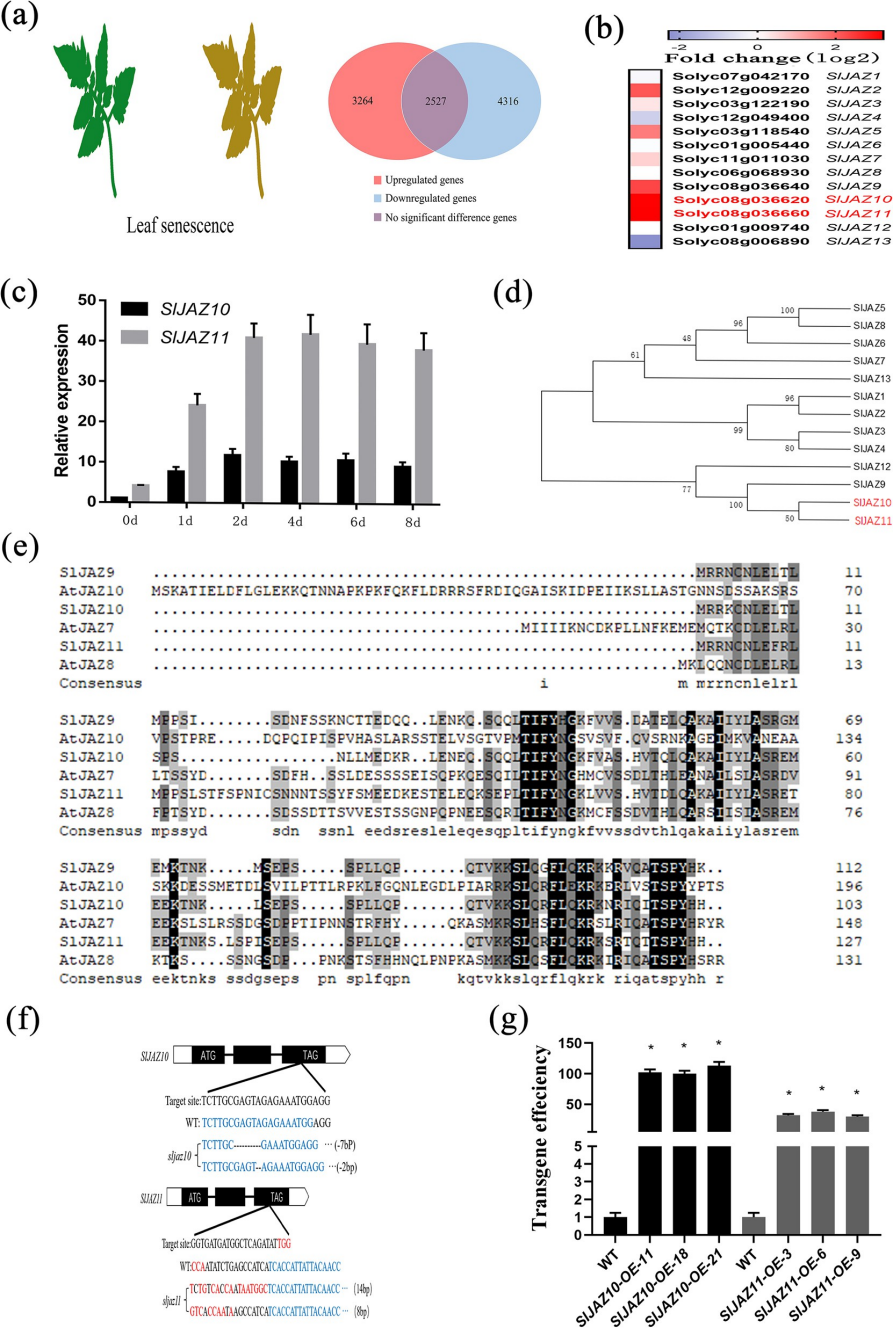
Expression of *SlJAZ10* and *SlJAZ11* is induced after dark treatment. (a) Distribution of genes upregulated or downregulated by dark treatment in the wild-type. (b) Expression of representative SlJAZ genes during aging. The fold-change in the average expression (log2 scale) of each gene is shown. WT samples were taken 8 days after dark treatment. Three independent biological samples were used. (c) The relative expression level of *SlJAZ10* and *SlJAZ11* during aging. (d) Phylogenetic analysis of JAZ proteins in tomato. (e) Amino acid sequence alignment of SlJAZ10 and SlJAZ11 homologues. (f) *sljaz10*^*ko*^ (CRISPR/Cas9-SlJAZ10) and *sljaz11*^*ko*^ (CRISPR/Cas9-SlJAZ11) alleles identified from the T1 mutants of tomato. (g) The *SlJAZ10*-overexpressing (OE) and *SlJAZ11*-overexpressing (OE) lines. The tissue examined was mature leaf.

To investigate the transcriptional regulation of the senescence of detached leaves in tomato, we treated WT tomato leaves with dark-induced senescence for 8 days. Following the dark-induced senescence treatment, the sequencing results showed a total of 7580 genes (3264 upregulated genes and 4316 downregulated genes) were significantly different (Fig 1A). Further analysis of the JAZ family of transcriptome data revealed that many JAZ genes could respond to the senescence of detached leaves (Fig 1B). Here, *SlJAZ10* and *SlJAZ11* were the two most significantly induced genes by senescence among 13 JAZ. To further validate the transcriptome data, we analysed the senescence expression pattern of *SlJAZ10* and *SlJAZ11* (Fig 1C). The transcript levels of *SlJAZ10* and *SlJAZ11* always remained at high levels over time. We then constructed a phylogenetic tree of the JAZ family in tomato (Fig 1D). Homology analysis showed that SlJAZ10 and SlJAZ11 shared high homology in protein sequence. Sequence alignments revealed that SlJAZ10 and SlJAZ11 share the highest similarities with Arabidopsis AtJAZ7 and AtJAZ8, respectively (Fig 1E). These observations provided supporting evidence that SlJAZ10 and SlJAZ11 presumably played similar roles in the senescence of detached leaves.

### SlJAZ10 and SlJAZ11 were involved in the senescence of detached leaves

**Fig 2:**
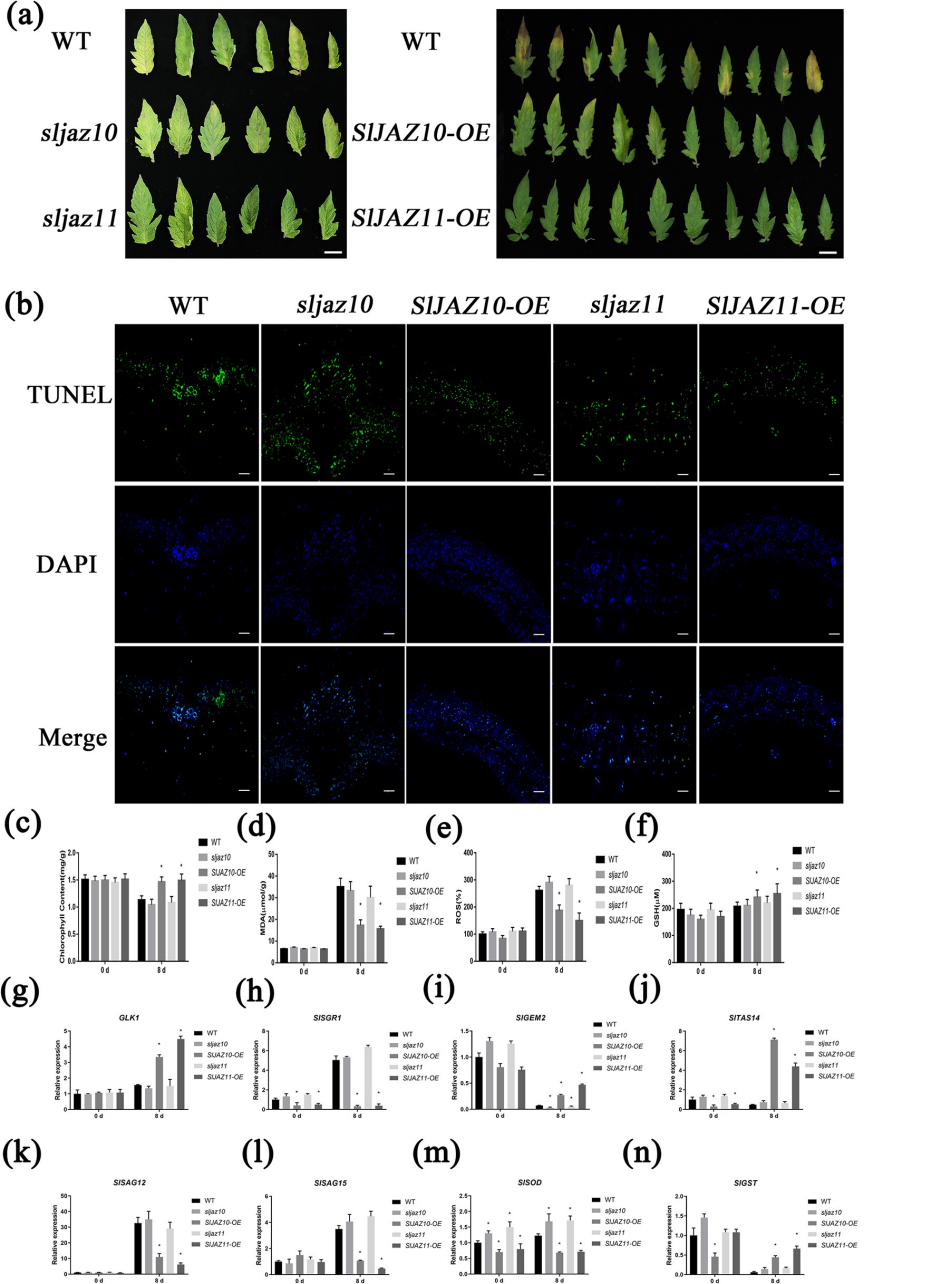
Physiological analyses of the mature leaves in tomato wild-type (WT), knockdown lines and overexpression lines after 8 days dark treatment. (a) Mature leaves of the wild type, knock-out, and overexpression lines under dark treatment, incubated with wetted filter paper in dark conditions for 8d, and photographed. (b) Cell death was detected using the Tunel assay. Representative images show Tunel staining results (blue, DAPI; green, Tunel). (c-f) Contents of chlorophyll, MDA, ROS and GSH in the leaves of the WT, *sljaz10*, *SlJAZ10*-OE, *sljaz11* and *SlJAZ11*-OE after dark treatment. (g-n) Relative expression of eight genes related to the above physiological phenotypes. All data are means (±SE) of three independent biological replicates. *P<0.01 (significant difference between mutants and WT according to Student^`^s t-test).

Because of the high expression levels of *SlJAZ10* and *SlJAZ11* during leaf ageing, we generated knockout lines by the CRISPR/Cas9 technique and overexpression lines by the CaMV 35S promoter. From the transgenic progeny, we selected two homozygous knockout lines (*sljaz10* and *sljaz11* one for each) (Fig 1F) and three overexpression lines with high expression levels of *SlJAZ10* and *SlJAZ11*, respectively, for phenotypic analysis (*SlJAZ10-OE-11*, *SlJAZ10-OE-18*, *SlJAZ10-OE-21*, *SlJAZ11-OE-3*, *SlJAZ11-OE-6* and *SlJAZ10-OE-9*) (Fig 1G). Compared with the WT (Fig 2A), *sljaz10* and *sljaz11* showed obvious yellowing at approximately the same time (8 d dark treatment). In contrast to the knockout lines, *SlJAZ10-OE* leaves displayed little chlorotic symptoms and *SlJAZ11-OE* leaves almost did not display chlorosis, while WT leaves showed obvious chlorotic symptoms in the ageing phase (8 d dark treatment).

To further substantiate the above results, we performed biochemical and qRT–PCR analysis of the samples with dark-induced leaf senescence. TUNEL assay showed that the ratios of dead to live cells of *sljaz10* and *sljaz11* indistinguishable from the WT were observed and the cell survival rate of *SlJAZ10-OE* and *SlJAZ11-OE* was higher than the WT (Fig 2B). Measurement of chlorophyll content showed that chlorophyll loss was much slower in the leaves of the overexpression lines (*SlJAZ10-OE* and *SlJAZ11-OE*) than in the wild type and in knockout lines (*sljaz10* and *sljaz11*) under dark treatment (Fig 2C). Correspondingly, MDA content was significantly lower in both overexpression lines than in the WT and knockout lines (Fig 2D). Given that the accumulation of mitochondrial ROS and GSH has been linked to cellular senescence [42], we performed measurements on all samples. After dark treatment, the ROS content of the two overexpression lines was significantly lower than that in the WT and knockout lines (Fig 2E), and the GSH content in the overexpression lines greatly increased than that in the WT and knockout lines (Fig 2F). The survival rate results were consistent with the biochemical results reported above (S1 Fig). To verify the accuracy of the biochemical indicators, eight transcriptional regulatory genes (*SlGLK1*, *SlSGR1*, *SlTAS14*, *SlGME2*, *SlSAG12*, *SlSAG15*, *SlSOD* and *SlGST*) involved in the physiological indices mentioned above were selected for quantification. As anticipated, these consequences of quantitative analysis were consistent with the biochemical parameters during leaf ageing (Fig 2G–2N). In summary, these results suggest that knockout of *SlJAZ10* or *SlJAZ11* did not affect the antiaging effect of the transgenic plant leaves, but it could enhance the antiaging effect by overexpressing *SlJAZ10* or *SlJAZ11*, respectively.

### *SlJAZ10* and *SlJAZ11* regulate regeneration through JA signalling

**Fig 3:**
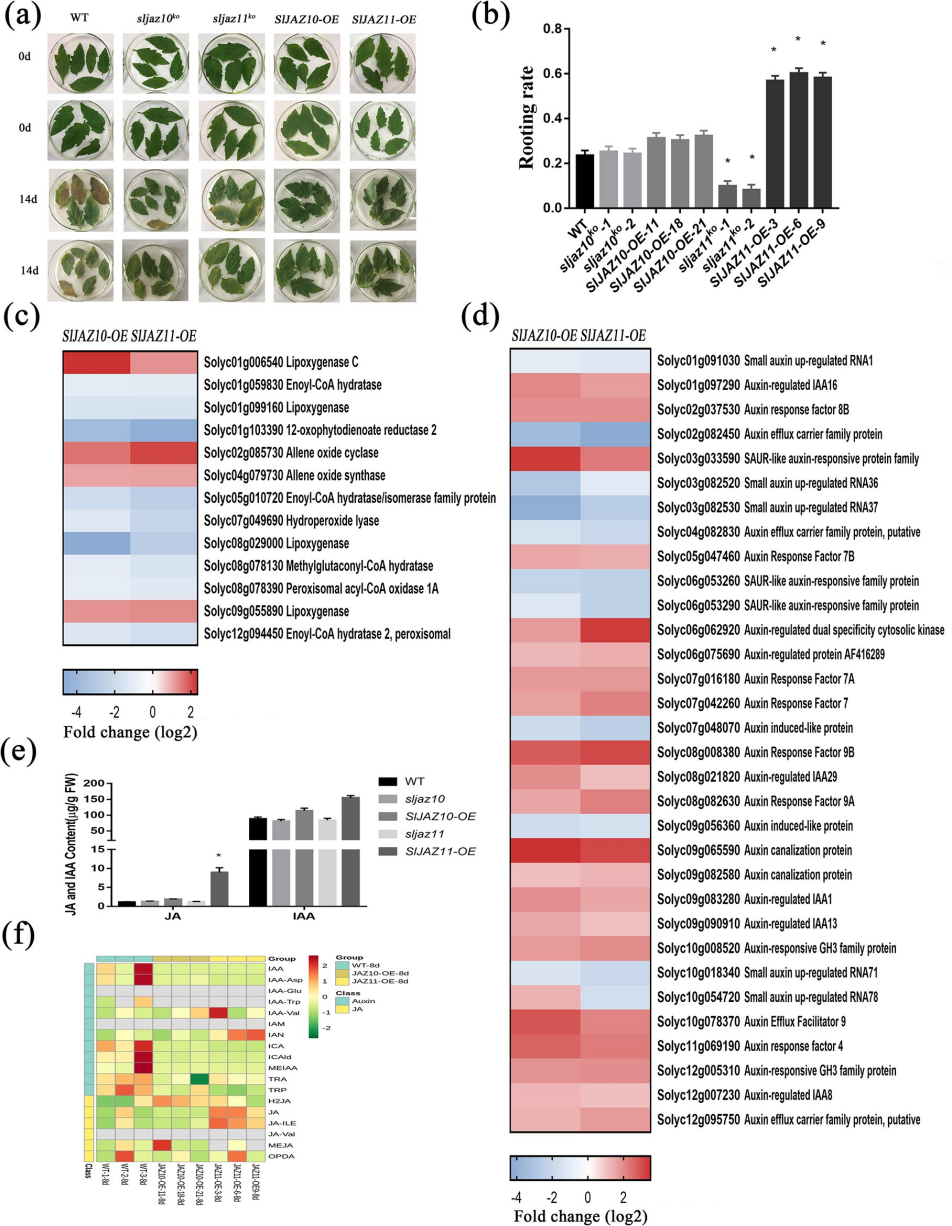
*SlJAZ10* and *SlJAZ11* regulate JA and IAA signaling in dark-induced senescence. (a) Mature leaves of the wild type and transgenic lines under dark treatment, incubated with wetted filter paper in dark conditions for 14d, and photographed. (b) The rooting rate of the wild type and transgenic lines under 14d dark treatment. *P<0.01 (significant difference between mutants and WT according to Student^`^s t-test). (c) Expression of JA-responsive related genes in the RNA-seq experiments. The fold-change in the average expression (log2 scale) of each gene is shown. (d) Expression of IAA-responsive related genes in the RNA-seq experiments. The fold-change in the average expression (log2 scale) of each gene is shown. (e) JA content and IAA content of senescent leaves. (f) Heatmaps of hormone metabolism. The heat maps represent the log2 fold changes of DEGs related to hormone metabolisms.

To probe the antiaging limits of *SlJAZ10-OE* and *SlJAZ11-OE* transgenic lines, we prolonged the dark-treatment from 8 to 14 days. Although many transgenic line leaves began to exhibit senescence, they did better than WT leaves. Quite unexpectedly, some stay-green leaves formed calli and generated roots at the petiole (Fig 3A). For statistical analysis, both the rooting rate of *sljaz10*^*ko*^ and *SlJAZ10-OE* transgenic plants was indistinguishable from WT, but overexpression of SlJAZ11 could dramatically promote rooting (Fig 3B).

Subsequently, we performed transcriptome sequencing analysis of dark-treated leaf samples after 8 days. Compared with wild-type leaves, differential gene expression analysis revealed that 4791 and 5732 genes were affected in *SlJAZ10-OE* and *SlJAZ11-OE* transgenic lines, respectively, during dark-treatment (S2A Fig). The DEGs of the two transgenic plants were further refined through Gene Ontology (GO) enrichment and KEGG pathway enrichment analyses (S2B and S2C Fig), and the results showed that *SlJAZ10* and *SlJAZ11* could coordinate primary and secondary metabolites to resist to senescence. A large number of DEGs associated with the IAA and JA signalling pathways were identified in both *SlJAZ10-OE* and *SlJAZ11-OE* lines (Fig 3C and 3D). After measuring the JA and IAA contents of untreated samples, we revealed that only the JA content of *SlJAZ11-OE* was significantly upregulated (Fig 3E). The results of hormone metabolism analysis indicated that only JA and JA-Ile contents were upregulated significantly in the *SlJAZ11-OE* transgenic lines after dark-treatment (Fig 3F). To further validate the effects of hormones on the *sljaz10*, *SlJAZ10-OE*, *sljaz11* and *SlJAZ11-OE* transgenic lines, we further treated seedlings with JA biosynthetic inhibitor ibuprofen (IBU) and auxin transport inhibitor (TIBA) (S3 Fig). When compared with the wild-type, all transgenic lines (*sljaz10*, *SlJAZ10-OE*, *sljaz11* and *SlJAZ11-OE*) were less sensitive to IBU and were not significantly different with TIBA treatment. The *SlJAZ11-OE* transgenic lines showed better root development than *SlJAZ10-OE* transgenic lines with IBU treatment. The joint analysis of hormone and transcriptomics data and its responsiveness to JA suggested that the JA signalling pathway was a critical factor in antiaging and regeneration.

### JJW (SlJAZ10-SlJAV1-SlWRKY51 or SlJAZ11-SlJAV1-SlWRKY51) binds and represses JA biosynthesis gene (*SlAOC*)

**Fig 4:**
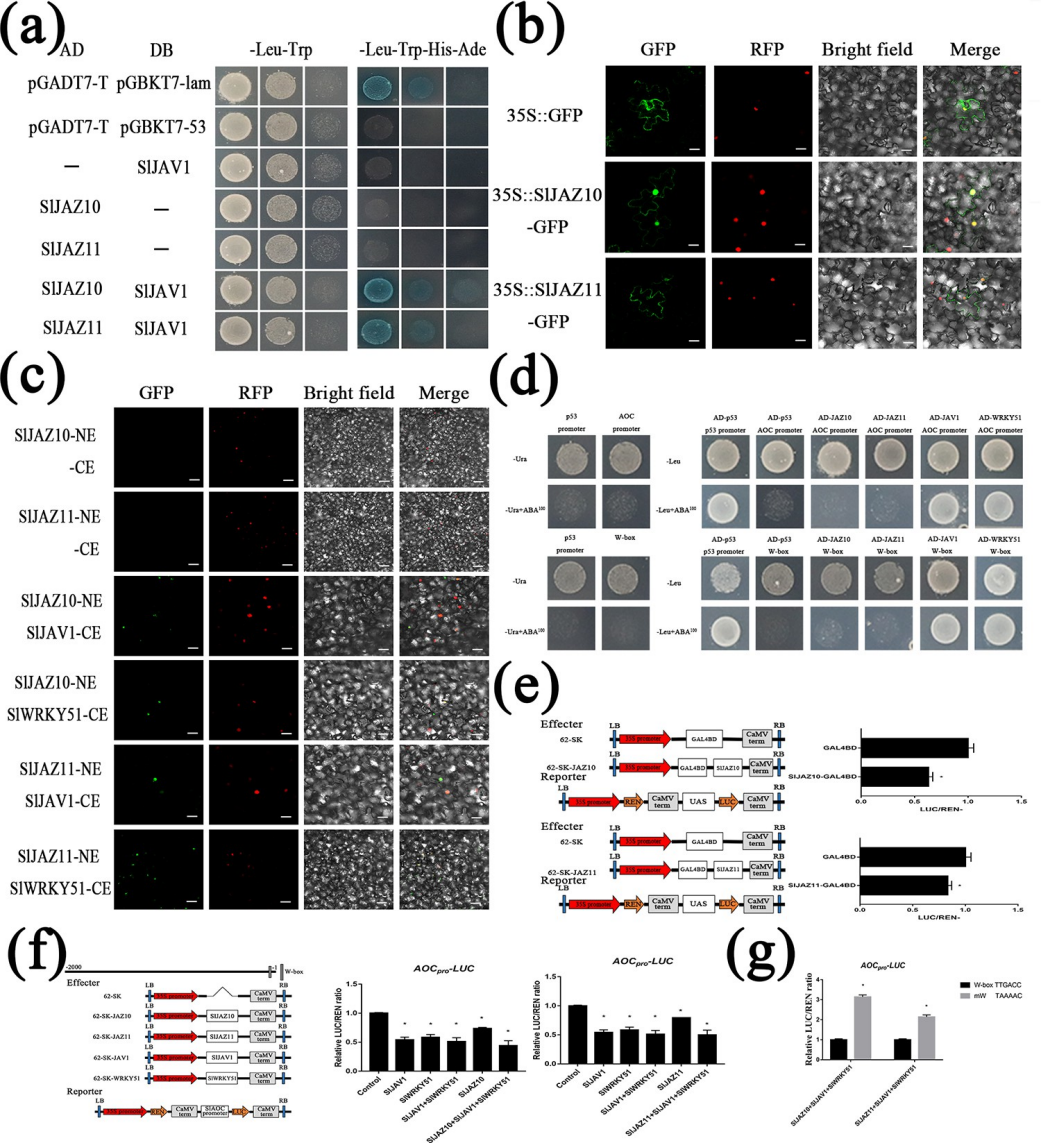
The JJW (SlJAZ10-SlJAV1-SlWRKY51 or SlJAZ10-SlJAV1-SlWRKY51). (a) Y2H assay shows that SlJAV1 interacts with SlJAZ10 and SlJAZ11. (b) Subcellular localization of SlJAZ10 and SlJAZ11 in the epidermal cells of *N*. *benthamiana* leaves. Bars = 40μm. (c) BiFC assay confirms the interactions among SlJAZ10, SlJAZ11, SlJAV1 and SlWRKY51 in *N*. *benthamiana* leaves. (d) Y1H assay shows the transcriptional binding activity of SlJAV1 and SlWRKY51 with *SlAOC* promoter. (e) Transient transcriptional activation assays show that SlJAZ10 and SlJAZ11 have transcriptional repression activity. (f) SlJAZ10-SlJAV1-SlWRKY51 and SlJAZ11-SlJAV1-SlWRKY51complex effectively suppresses the expression of *SlAOC*_Pro_*-LUC* in *N*. *benthamiana* transient expression assay. Relative ratio of LUC/REN are means ± SEM (n≥6); *p < 0. and *p < 0.05; Student’s t test. (g) Relative expression of the luciferase gene (LUC) driven by the 130bp upstream fragment of the *SlAOC* promoter, with the W-box intacted or mutated.

In previous studies, JAV1, JAZ8 and WRKY51 were shown to form a corepressor complex to bind and repress JA biosynthesis genes in *Arabidopsis* [37]. Y2H screening showed that SlJAV1 interacts with SlJAZ10 and SlJAZ11 (Fig 4A). However, toxicity testing indicated that the full-length SlWRKY51 protein caused toxic effects in the yeast host cell, so there was no Y2H assay for SlWRKY51. The subcellular localization assay showed that SlJAZ10 and SlJAZ11 were associated with the nucleus and membrane (Fig 4B). Further BIFC and Co-IP assays detected physical interactions among SlJAZs (SlJAZ10 or SlJAZ11), SlJAV1, and SlWRKY51 in vivo (Figs 4C and S4).

In *Arabidopsis*, the JAV1-JAZ8-WRKY51 complex directly bound to the W-box of the *AOS* promoter (JA biosynthesis gene) [43]. Subsequent analysis showed that the W-box motif was present in the promoters of *SlAOC* (JA biosynthesis gene) and *SlAOS* (JA biosynthesis gene). For this purpose, we used specific primers surrounding the W-box of the *SlAOC* and *SlAOS* promoters for the Y1H assay (Figs 4D and S5). Unexpectedly, the SlJAV1 and SlWRKY51 proteins could bind to the promoter of *SlAOC* and W-box but not to the promoter of *SlAOS*, while SlJAZ10 and SlJAZ11 could not bind to the W-box motif in the promoter of *SlAOC* and *SlAOS*.

Subsequently, transcriptional activity experiments indicated that the SlJAZ10 and SlJAZ11 proteins had transcriptional repressive activity (Fig 4E). The JJW complex did not enhance the suppression of SlJAZ10 and SlJAZ11 (S6 Fig). To evaluate the effect of SlJAZ10, SlJAZ11, SlJAV1 and SlWRKY51 on *SlAOC* and *SlAOS* expression, the promoters of *SlAOC* and *SlAOS* were fused with the luciferase gene to generate the reporters, whereas SlJAZ10, SlJAZ11, SlJAV1 and SlWRKY51 driven by the CaMV 35S promoter were used as effectors (Figs 4F and S7A). As shown, both individual proteins in all reporters and the JJW protein complex significantly repressed the activity of *SlAOC*_*Pro*_*-LUC*. However, none of the effectors regulated the activity of *SlAOS*_*Pro*_*-LUC* (S7B Fig). Consistent with the dual-luciferase assay, quantitative PCR analysis showed that only *SlJAZ11* could regulate the expression of *SlAOC* (S7C Fig). Enhanced binding of JJW was observed upon mutation of the W-box (Fig 4G). Interestingly, although both of the *SlAOC* and *SlAOS* promoters contain W-box motifs, only the promoter of *SlAOC* was regulated by JJW.

### Injury-triggered regeneration in transgenic tomato plants

**Fig 5:**
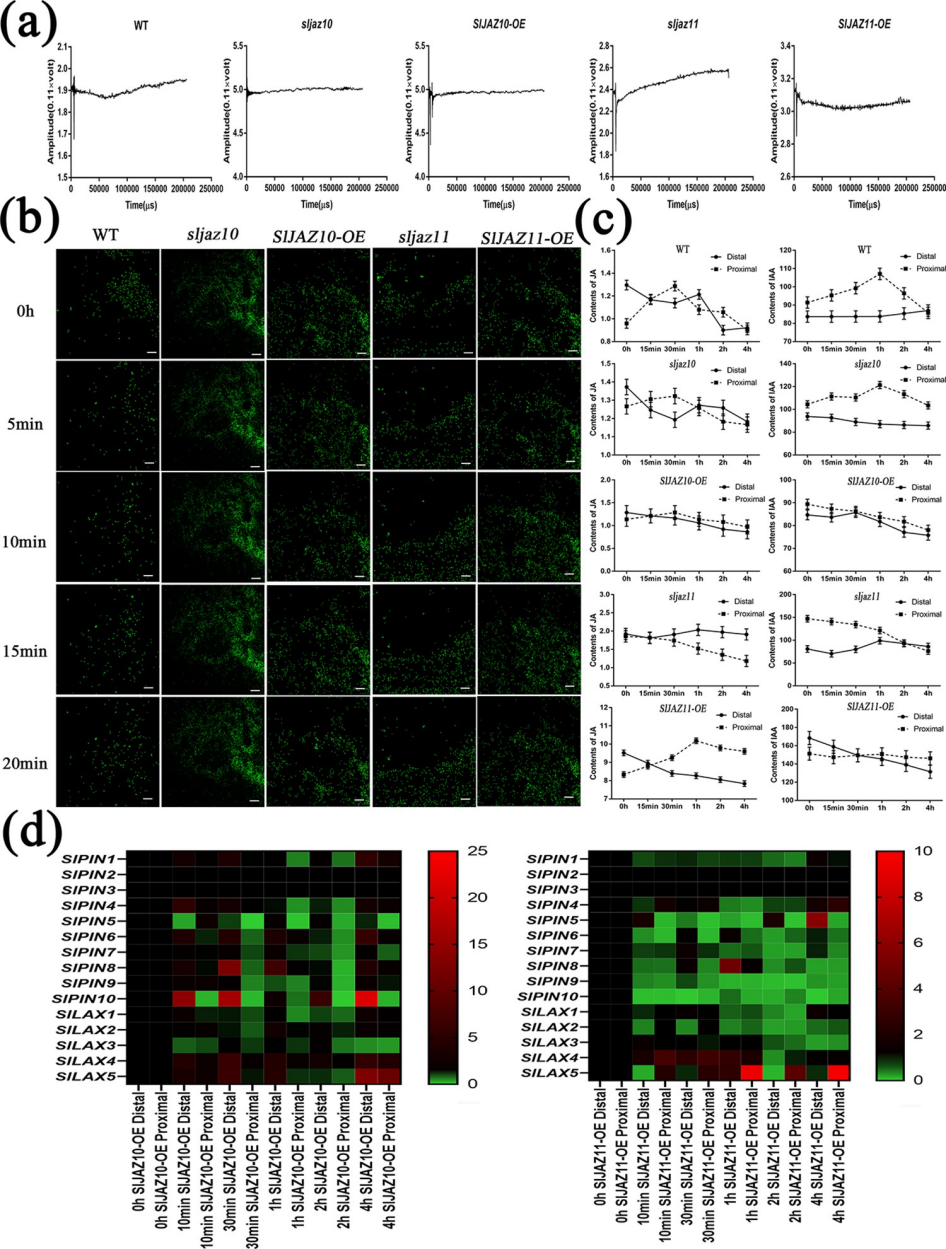
Changes in electrical signals, calcium signals and hormone transports during injury-induced regeneration. (a) Typical WASPs in wild type (WT), *sljaz10*, *SlJAZ10-OE sljaz11* and *SlJAZ11-OE* after wounding leaves. (b) Confocal image of [Ca^2+^]_cyt_ in WT, *sljaz10*, *SlJAZ10-OE sljaz11* and *SlJAZ11-OE* after wounding leaves. (c) Relative contents of JA and IAA at both ends of the wounding leaves. (d) Heatmap of auxin transport unigenes at both ends of the wounding leaves.

Because many regulators of electrical signals and calcium (Ca^2+^) waves were heavily enriched in the transcriptome data of dark-treated samples (Table 1), we next focused our work on the changes in electrical signals and Ca^2+^ waves after wounding. The surface potential from the proximal wound area to the proximal wound area was measured from damage initiation to 20 min. The results of wound-activated surface potential changes (WASPs) of variable amplitude are shown in Fig 5A. For the periods of time after wounding, the electrical signal of WT was first transmitted from the distal ends to the wound and then transmitted to the distal ends. For *SlJAZ10*, the electrical signal of both knockdown and overexpression transgenic lines was virtually unchanged after wounding. However, the electrical signal of the *sljaz10* transgenic line consistently transported away from the wound while the electrical signal of the *SlJAZ11-OE* transgenic line throughout converged towards the wound. Similar to the WSAP results after wounding (Fig 5B), the wave of Ca^2+^ movement in WT, *sljaz10* and *SlJAZ10-OE* was disorganized, while the movement of Ca^2+^ in the *sljaz11* transgenic line moved from the wound to the distal ends and in the *SlJAZ11-OE* transgenic line, it converged towards the wound.

**Table 1:** Differential genes expression analysis.

#	Locus Identifier	logFC (SlJAZ10)	PValue (SlJAZ10)	LogFC (SlJAZ11)	PValue (SlJAZ11)	Annotation
1	Solyc01g008130	-1.7671874	2.02E-43	-2.975199912	2.31E-82	Electron protein, putative (Protein of unknown function, DUF547)
2	Solyc02g089980	-	-	1.250324751	3.30E-18	Electron transporter, putative (Protein of unknown function, DUF547)
3	Solyc04g082970	-	-	2.12014617	1.42E-86	Electron protein, putative (Protein of unknown function, DUF547)
4	Solyc08g078520	1.757863942	3.91E-07	1.716670736	3.13E-06	Electron transporter, putative (Protein of unknown function, DUF547)
5	Solyc09g089880	-1.5977942	2.50E-67	-2.385733953	3.34E-120	Electron transfer flavoprotein beta-subunit, putative
6	Solyc12g005200	-2.902997529	3.74E-283	-3.455028482	5.92E-318	Electron transfer flavoprotein alpha subunit
7	Solyc01g007170	1.847217662	1.44E-05	2.116576732	3.83E-06	C2 calcium/lipid-binding plant phosphoribosyl transferase family protein
8	Solyc01g065500	1.811816639	3.01E-33	2.64713281	4.25E-75	C2 calcium/lipid-binding plant phosphoribosyl transferase family protein
9	Solyc01g073710	-1.133565739	1.34E-14	-1.987680422	4.56E-33	Calcium-binding EF hand family protein
10	Solyc01g091465	3.366723725	2.28E-08	3.77719715	8.27E-11	Calcium-binding EF-hand
11	Solyc01g097420	-2.079749473	7.31E-14	-3.412797764	1.55E-21	Calcium-transporting ATPase
12	Solyc01g099385	-2.203688611	1.92E-10	-1.680613943	7.33E-07	Calcium-dependent lipid-binding (Ca LB domain) family protein
13	Solyc01g104150	1.414469094	1.25E-04	2.209209922	5.25E-10	Calcium ion-binding protein
14	Solyc01g108190	-1.289510585	9.15E-58	-2.354040652	1.85E-153	Calcium-binding EF-hand
15	Solyc02g065555	-1.672617962	5.53E-75	-1.647949923	2.36E-78	Calcium uniporter, mitochondrial
16	Solyc02g079520	2.646693288	1.12E-63	3.562434818	2.66E-159	Calcium-binding EF-hand protein
17	Solyc02g083850	-1.80201821	4.92E-60	-2.760931591	1.53E-88	Calcium-dependent protein kinase
18	Solyc02g088090	1.014703362	5.13E-23	2.153781727	3.11E-111	Calcium-binding EF-hand
19	Solyc02g090560	2.607959043	1.63E-16	2.162216007	9.53E-09	Calcium-transporting ATPase
20	Solyc03g006260	3.540268625	2.08E-24	2.680131648	8.61E-09	Calcium-binding EF-hand
21	Solyc03g044960	-5.423833409	1.42E-209	-7.181839469	8.39E-213	Calcium uniporter protein, mitochondrial
22	Solyc04g007800	1.800590518	2.16E-07	1.514884396	1.92E-04	Calcium-dependent lipid-binding (Ca LB domain) family protein
23	Solyc04g015070	1.927481861	3.04E-17	2.374922391	1.31E-27	Calcium-dependent lipid-binding (Ca LB domain) family protein
24	Solyc04g058160	-1.406953835	1.87E-20	-2.984968019	5.16E-46	Calcium-binding protein
25	Solyc04g058170	-3.527497763	1.03E-29	-3.500188831	4.16E-25	Calcium-binding protein
26	Solyc07g045210	1.248666809	2.56E-38	1.388869303	3.84E-47	Calcium ion-binding protein
27	Solyc08g008020	1.627514206	6.48E-08	2.564625232	5.85E-18	C2 calcium/lipid-binding plant phosphoribosyl transferase family protein
28	Solyc09g007860	-3.061286993	1.67E-34	-3.675206534	9.11E-38	Calcium-dependent lipid-binding (Ca LB domain) family protein
29	Solyc10g006740	2.907308454	6.29E-10	3.229458205	5.95E-12	Calcium-binding family protein
30	Solyc10g050060	-1.638049538	1.24E-72	-1.503471306	1.47E-62	Calcium-dependent lipid-binding (Ca LB domain) family protein
31	Solyc10g074570	-1.575154962	6.59E-72	-2.027645716	3.37E-120	Calcium-dependent protein kinase family protein
32	Solyc10g078680	4.587793038	2.39E-66	4.803329662	6.82E-76	C2 calcium/lipid-binding and GRAM domain protein
33	Solyc10g080420	1.101188016	8.14E-34	1.498154783	1.77E-64	C2 calcium/lipid-binding and GRAM domain protein
34	Solyc11g018610	-1.157052799	4.19E-12	-1.019775783	5.83E-09	Calcium-dependent protein kinase
35	Solyc11g022460	1.194261168	1.20E-26	1.488195894	3.26E-36	C2 calcium/lipid-binding plant phosphoribosyl transferase family protein
36	Solyc11g068460	-1.915436535	1.12E-78	-1.106358978	6.89E-34	Ef-hand calcium binding protein, putative
37	Solyc12g011420	-1.119432794	2.12E-12	-2.4207635	4.94E-33	Calcium-dependent lipid-binding domain protein
38	Solyc12g014110	1.287057008	1.45E-32	1.796354305	7.64E-66	Sodium/calcium exchanger family protein / calcium-binding EF hand family protein
39	Solyc12g088840	2.54541436	7.85E-82	2.056328816	3.20E-34	Calcium-binding EF-hand family protein, putative

In *Arabidopsis*, the regulation of JA and IAA was accomplished by the rapid response electrical signal and Ca^2+^ wave [44, 45]. Therefore, we measured the changes in the JA and IAA contents at the two ends of the wound (Fig 5C). Following injury, the total content of JA and IAA showed a gradually decreasing trend in all samples. An unexpected observation was that the relative content of JA and IAA of the two ends of the wound had a certain regularity in its spread. Immediately after injury, the contents of JA and IAA (WT, sljaz10 and SlJAZ10-OE) were actively transported into the approximate wound boundary, and they were transported further away from the wound after a while. In the *sljaz11* transgenic line, JA and IAA were slowly transported from the proximal ends to the distal ends. In contrast to the other transgenic, JA and IAA were concentrated close to the wound in the *SlJAZ11-OE* transgenic line.

By qRT–PCR, we found that many auxin transport-related genes displayed significant changes at the two ends of the wound (Fig 5D). In *SlJAZ10-OE* and *SlJAZ11-OE* transgenic lines, these auxin transport-related genes were induced to respond to injury upon mechanical wounding. Overall, different from the regulation of *SlJAZ10* after wounding, the regulation of *SlJAZ11* was performed towards the wound ends.

### Ca^2+^ and SlRbcs-3B control JJW-regulated JA biosynthesis for regeneration

**Fig 6:**
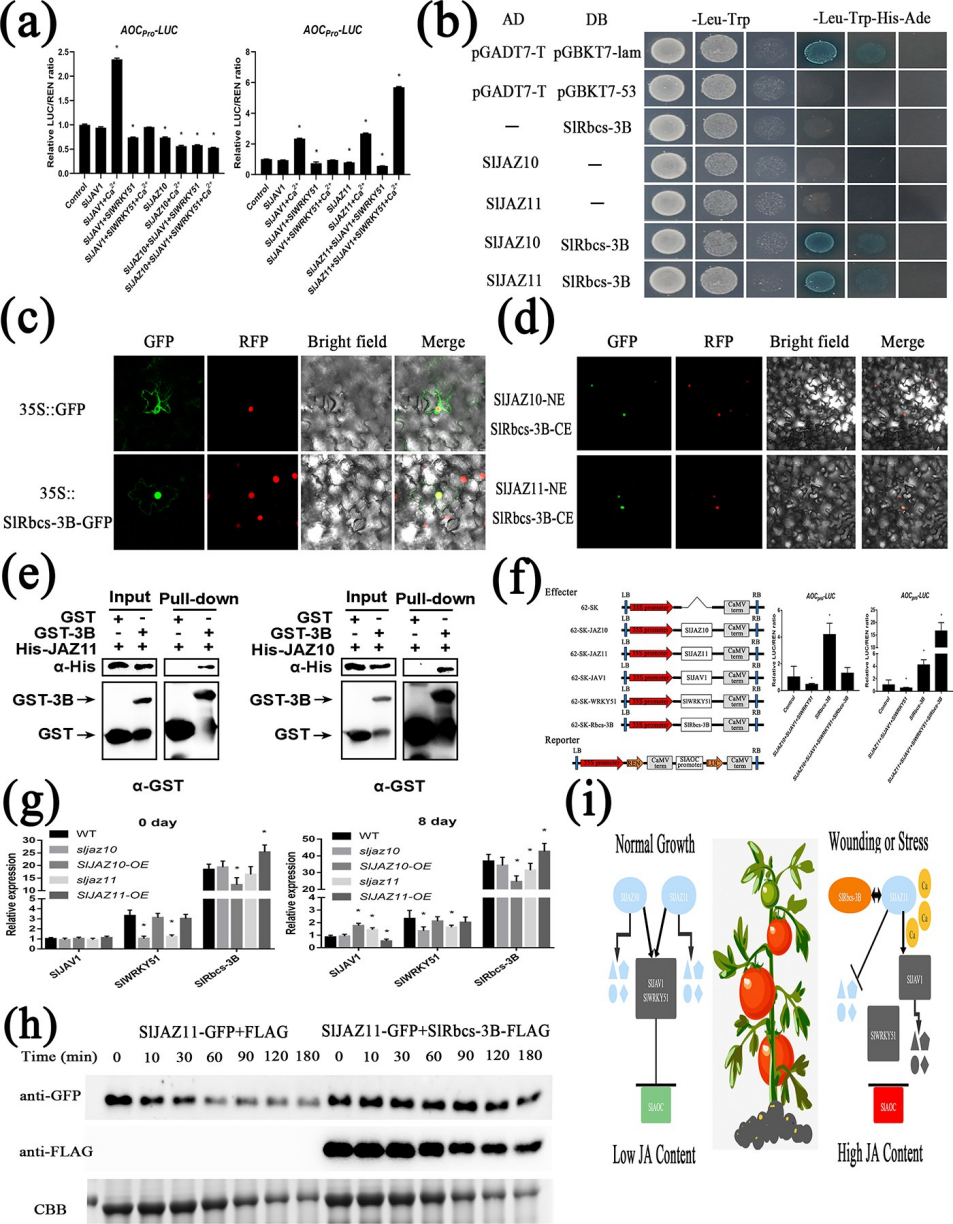
Ca^2+^ and SlRbcs-3B inactivates JJW Complex. (a) Ca^2+^ alter transcriptional repression of SlJAZ10-SlJAV1-SlWRKY51 and SlJAZ11-SlJAV1-SlWRKY51 complex on the *SlAOC*_*Pro*_*-LUC* expression. Relative ratio of LUC/REN are means ± SEM (n≥6); *p < 0.01; Student’s t test. (b) Y2H assay shows that SlRbcs-3B interacts with SlJAZ10 and SlJAZ11. (c) Subcellular localization of SlRbcs-3B in the epidermal cells of *N*. *benthamiana* leaves. Bars = 40μm. (d) BiFC assay confirms the interactions among SlJAZ10, SlJAZ11, and SlRbcs-3B in *N*. *benthamiana* leaves. (e) The interaction of His-SlJAZ10 and His-SlJAZ11 with GST-SlRbcs-3B detected by the pull-down assay. GST-SlRbcs-3B was used as bait, and pull-down of His-SlJAZ10 and His-SlJAZ11 was detected by anti-His antibody. (f) SlRbcs-3B alters transcriptional repression of SlJAZ11-SlJAV1-SlWRKY51 complex on the *SlAOC*_*Pro*_*-LUC* expression. Relative ratio of LUC/REN are means ± SEM (n≥6); *p < 0; Student’s t test. (g) qRT-PCR analysis of transcript levels of *SlJAV1*, *SlWRKY51* and *SlRbcs-3B* in dark-treatment leaves of WT, *sljaz10*, *SlJAZ10-OE*, *sljaz11* and *SlJAZ11-OE* plants. Data are means ± SEM (n≥3); *p < 0.01; Student’s t test. (h) SlRbcs-3B promotes maintenance the stabilization of SlJAZ11 proteins in vivo. Proteins were extracted form *N*. *benthamiana* leaves transiently expressing SlJAZ11-GFP or SlRbcs-3B-Flag alone. Extracts containing SlRbcs-3B-Flag were incubated with SlJAZ11-GFP or GFP extracts for different times. Degradation of SlJAZ11-GFP was detected by anti-GFP antibody. An equal amount of SlRbcs-3B-Flag or Flag was detected by anti-Flag antibody. The equal amount of protein stained by CBB was used as a loading control. (i) A simplified model for injury-triggered JA biosynthesis in plant defense.

The calcium ions efficiently degraded JAZ-JAV-WRKY protein complexes by phosphorylation of JAV1 [37]. Given the injury experiment results, we investigated the effects of Ca^2+^ on JJW activity (Fig 6A). Coexpression of Ca^2+^ and SlJAV1 obviously promoted *SlAOC*_*Pro*_*-LUC* expression compared to SlJAV1 alone. Moreover, the repression of SlJVA1-SlWRKY51 complex activity was abolished by Ca^2+^. Interestingly, after the addition of Ca^2+^, the transcriptional function of SlJAZ11 or the SlJAZ11-SlJAV1-SlWRKY51 complex transforms the repression to activation of *SlAOC*. However, Ca^2+^ did not change the repression of the SlJAZ10 and SlJAZ10-SlJAV1-SlWRKY51 complex. All correlation changes occurred only for *SlAOC* but not for *SlAOS* (S8 Fig).

To gain further insights into the molecular functions of SlJAZ10 and SlJAZ11, we searched for interacting proteins by yeast two-hybrid screening. SlRbcs-3B (ribulose bisphosphate carboxylase small chain), a photosynthesis gene, was identified by yeast two-hybrid screening (Fig 6B). In agreement with the literature report [46], the analysis of subcellular localization showed that SlRbcs-3B was localized in the nucleus (Fig 6C). The interaction in the Y2H assay was further validated by BiFC (Fig 6D) and pull-down assays (Fig 6E).

To figure out the function of SlRbcs-3B in JA biosynthesis for regeneration, we focused on its regulation of JJW complexes. The experiments with a dual-luciferase-reporter indicated that SlRbcs-3B may not only drastically induce the expression of *SlAOC* promoters but also switch the activity of SlJAZ11-SlJAV1-SlWRKY51 from inhibition to promotion (Fig 6F). Real-time PCR of senescent samples revealed that the expression of *SlJAV1*, *SlWRKY51* and *SlRbcs-3B* was induced to varying degrees by *SlJAZ10* and *SlJAZ11* during leaf senescence (Fig 6G). From the above protein experiments, we also found that SlJAZ11 proteins might be degraded rapidly. In vitro protein degradation assays (Fig 6H) demonstrated that the SlJAZ11 protein was indeed susceptible to degradation. SlRbcs-3B might promote the stabilization of SlJAZ11 protein within a certain period of time. Collectively, Ca^2+^ and SlRbcs-3B could alter the regulatory function of JJW to regulate regeneration.

## Discussion

Senescence is a multifactorial process integrating hormonal and molecular perturbations. JA plays an important role in plant senescence and defence [47]. Both natural and dark-induced senescence could lead to high JA levels [48]. By suppressing transcription factors, JAZs maintain JA at low levels to repress the expression of JA-responsive genes [49]. However, JAZs are rapidly degraded upon high JA levels, which can release and trigger JA signalling pathway activation [50]. In *Arabidopsis*, JAZ7-mediated MYC-regulated indole-GS genes are involved in leaf senescence [6], and JAZ8 is a component of the inhibitory JA signalling pathway that could lead to the inhibition of plant growth and reduce plant defence [51]. Our phenotypic observations of transgenic lines are consistent with these data and further support the regulatory mechanisms of leaf senescence through the JAZ signalling pathway. The *SlJAZ10* and *SlJAZ11* overexpression transgenic lines strongly enhanced the ability to resist dark-induced senescence (Fig 2) by JA-mediated signalling pathways (Fig 3). We found that *SlJAZ10* and *SlJAZ11* repressed ageing related genes to negatively regulate leaf senescence, agreeing with previous findings [51]. JA is known to promote dark-induced senescence [20], however, such a high content of JA in *SlJAZ11* overexpression transgenic lines is not consistent with previous research. JA sensitivity analysis (S9 Fig) indicated that *SlJAZ10* and *SlJAZ11* could actively repress the JA-responsive pathway. Additional JA treatment showed that *SlJAZ10* and *SlJAZ11* overexpressing transgenic lines could still keep anti-ageing at high JA levels through the regulation of the JA response.

It has long been known that high levels of JA are harmful to normal plant growth and development [52–54]. We note that such a model in which the JAV1-JAZ8-WRKY51 (JJW) complex binds and represses JA biosynthesis genes was proposed to repress the JA biosynthesis in *Arabidopsis* [37]. This yield is largely consistent with our experimentally observed results. During the course of studies, we found that *SlJAZ10* and *SlJAZ11* were expressed at lower levels and that SlJAZ10 and SlJAZ11 were susceptible to degradation under normal conditions. When plants respond to JA, JAZ proteins are recruited by COI1 to degradation [21, 49]. Taken together, our experiments showed that the SlJAV1-SlWRKY51 complex (JW complex) negatively regulated JA biosynthesis by direct binding of the W-box of the *SlAOC* promoter. These results of IBU-treated seedlings suggested that either knockout or overexpression of *SlJAZ10* or *SlJAZ11* increased JA sensitivity (S3 Fig). The inhibitory activity of the JW complex was lower than that of JJW of WT when *SlJAZ10* or *SlJAZ11* was knocked down and JAZ proteins repressed the transcription of JA-responsive genes when *SlJAZ10* or *SlJAZ11* was overexpressed. These data validate our hypothesis that *SlJAZ10* and *SlJAZ11* are not simply related to the JJW complex to regulate JA signalling pathway.

Given that JA content is normally present at low levels in normal plant growth, additional stimuli are required for the regulation of JA or JAZ protein mediated signalling. Although our research suggests some degree of functional redundancy between SlJAZ10 and SlJAZ11, they are much different especially for regeneration. Wounding is a prerequisite for plant regeneration, and the dynamic JA wave responds to wounding by rapidly influencing auxin signalling [19]. Here, we show that the difference in JA levels may be the major contributor to regeneration between *SlJAZ10-OE* and *SlJAZ11-OE*. However, prolonged treatment with JA did not further promote antiaging or regeneration (S9 Fig). This revealed that plant regeneration did not appear to be simply a JA-mediated plant defence response. Additional efforts, the moving direction of electrical signals and the flow of Ca^2+^ waves verified this hypothesis (Fig 5). Furthermore, we found that the movement of electrical signals took priority over the flow of Ca^2+^ waves after wounding. On the basis of previous studies and our current results, we suggest that SlJAZ11 responds preferentially to transport JA into the wound area by altering the electrical signals, and immediately following the Ca^2+^ wave, it also carries auxin to the wound margins in response to injury. Overall, the SlJAZ11 mediated plant regeneration is a complex process that involves electrical signals, Ca^2+^ waves, hormone metabolism, hormone transport and transcriptional regulators.

When plants are pressed by an external stimulus, the autoimmune defence mechanism rapidly activates the JA signalling pathway to induce the regulation of JA-related functions, leading to defence responses against the stress. However, since JAZ proteins are vulnerable to degradation, how JAZ proteins maintain stability and function remains to be elucidated. Ribulose bisphosphate carboxylase (Rubisco) is necessary for the survival of plants; thus, the robustness of the rbcS gene facilitates adaptation to drastic environmental changes or prevents loss of RBCS when some of the copies are lost. Although most studies of RBCS have focused on photosynthesis [55], some other published literature has shown that the expression of RBCS is affected by stress (ozone, drought, salt and metal) and wounding [56, 57]. Our studies indicate that SlRbcs-3B maintains the stability of the SlJAZ11 protein for a certain period of time. ERF109 (JA-responsive ethylene response factor 109) binds directly to the promoters of ASA1, and is well known to promote lateral root formation and mediates crosstalk between JA signalling and auxin biosynthesis [15]. Our study also suggested that SlJAZ11 regulated regeneration by stimulating of *SlERF109* and *SlASA1* expression (S10 Fig).

After years of related studies, the mechanism of JA-elicited plant defence has been shown for clarity, and the JA signalling pathway is becoming more detailed. However, jasmonat-zim-domain protein (JAZ) is well-known to researchers as a transcriptional repressor of JA signalling, and the mechanisms underlying the regulation of plant defence remain unknown. In this study, we have uncovered a new model of the molecular mechanism for the stress-induced burst of JA responses based on the results of the previous study (Fig 6I): during the period of normal growth in plants, SlJAZ10 and SlJAZ11 can promote the inhibitory activity of SlJAV1-SlWRKY51 (JJW) to repress JA biosynthesis genes, which subsequently enables the plant to grow and develop normally with low JA levels; when plants are stimulated, SlJAZ11 rapidly triggers the electrical signals and Ca^2+^ waves to degrade SlJAV1, which in turn activates degradation of the JJW complex to activate JA biosynthesis genes. This eventually leads to the rapid burst of JA that activates the plant defence response; furthermore, we also found that SlRbcs-3B could bind to stabilize SlJAZ11 for a period of time, and this interaction could protect SlJAZ11 to play an adaptive role over a long period of time in JA-induced regeneration.

## Supporting information

S1 Table Primers used for construction of transgenic vector. (DOCX)

S2 Table Primers used for qRT-PCR analysis. (DOCX)

S3 Table Primers used for construction of subcellular localization assay. (DOCX)

S4 Table Primers used for construction of Y2H vectors. (DOCX)

S5 Table Primers used for construction of Pull-down assay. (DOCX)

S6 Table Primers used for construction of Y1H. (DOCX)

S7 Table Primers used for construction of Vitro Degradation Assays. (DOCX)

S1 Fig Comparison of survival of WT and transgenic lines. (TIF)

S2 Fig Comparison of transcriptome data analysis of WT and transgenic lines. (a) Volcano plot for differential gene expression. (b) GO enrichment analysis. (c) KEGG and KEGG enrichment analysis of the DEPs.
(TIF)

S3 Fig Comparison of IBU and TIBA treatment of WT and transgenic lines. (TIF)

S4 Fig Co-immunoprecipitation (Co-IP) studies. (TIF)

S5 Fig Yeast one-hybrid analysis. (TIF)

S6 Fig Transcriptional activity assay of JJW. (TIF)

S7 Fig Dual luciferase reporter gene assay and quantitative PCR. (a) Binds the *SlAOC* and *SlAOS* promoter region. (b) SlJAZ10-SlJAV1-SlWRKY51 and SlJAZ11-SlJAV1-SlWRKY51complex effectively suppresses the expression of *SlAOS*_Pro_*-LUC* in *N*. *benthamiana* transient expression assay. (c) Relative gene expression of *SlAOC* and *SlAOS* in WT, *sljaz10*, *SlJAZ10*-OE, *sljaz11* and *SlJAZ11*-OE.
(TIF)

S8 Fig Ca^2+^ alter transcriptional repression of SlJAZ10-SlJAV1-SlWRKY51 and SlJAZ11-SlJAV1-SlWRKY51 complex on the *SlAOS*_*Pro*_*-LUC* expression. (TIF)

S9 Fig Comparison of JA treatment of WT and transgenic lines. (TIF)

S10 Fig Quantitative PCR of *SlERF109* and *SlASA1*. (TIF)
