# Supplementary material for: SlJAZ10 and SlJAZ11 mediate dark-induced leaf senescence and regeneration
Source: PLoS Genet. 2022 Jul 13;18(7):e1010285. doi: 10.1371/journal.pgen.1010285 (PMC9278786; doi:10.1371/journal.pgen.1010285)
Supplement: S1 Table — (DOCX) [file pgen.1010285.s001.docx]

**S1 Table.** Primers used for construction of transgenic vector

| Genes | Forward primer (5' to 3', top),  reverse primer (5' to 3', bottom) | Accession |
| --- | --- | --- |
| *sljaz10^ko^* | AAGTTATCTGAAATAGAAGGAGG  ACTGTGAAGAAATCTCTACAAGG | LOC101252609 |
| *SlJAZ10*-OE | GAAGAAAATGAGAAGAAAGTGT  AGTGATACACAAGTGTTGAGC | LOC101252609 |
| *sljaz11^ko^* | TCTTGCAAGTAGAGAAACAGAGG  GGTGATGATGGCTCAGATATTGG | LOC101253212 |
| *SlJAZ11*-OE | GCTTATGCCACCTTCTCTTTC  CACCACACGAGTAATGTTATCCTA | LOC101253212 |
