# Supplementary material for: SlJAZ10 and SlJAZ11 mediate dark-induced leaf senescence and regeneration
Source: PLoS Genet. 2022 Jul 13;18(7):e1010285. doi: 10.1371/journal.pgen.1010285 (PMC9278786; doi:10.1371/journal.pgen.1010285)
Supplement: S2 Table — (DOCX) [file pgen.1010285.s002.docx]

**S2 Table.** Primers used for qRT-PCR analysis

| Genes | Forward primer (5' to 3', top),  reverse primer (5' to 3', bottom) | Accession |
| --- | --- | --- |
| *SlJAZ10* | GGAACTCACTCTTTCTCCTAGCAAC  TGGTGATGAAGGCTCAGACAGCTT | LOC101252609 |
| *SlJAZ11* | GGAGTTTAGGCTTATGCCACCTTC  GGCTCAGATATTGGTGACAGACTC | LOC101253212 |
| *SlGLK1* | GAATTTTCCGTAAGCAGTGGTG  CTTCTCCTTGATTTAGGCTCGT | NM_001279264 |
| *SlSGR1* | ACTAGAAGGAAATGCAAGAAGAATCA  GCAACTTTCCTGGATGCTTTTC | NM_001247794 |
| *SlTAS14* | AGAAGGTGGGAGGAGAAAGAAG  ATGGAGATGAAAACAAAGGTGTT | NM_001247109 |
| *SlGME2* | CCATCACATTCCAGGACCAGA CGTAATCCTCAACCCATCCTTC | NM_001247660 |
| *SlSAG12* | TACCGGCCAACAACGAGAAG TGCAGCACCATATCCCACTG | LOC101251058 |
| *SlSAG15* | TTGGACGGGGAAATAAGGGC AAGATGGGCTGGAACCTTCG | LOC101248184 |
| *SlSOD* | TCCTGAAGATGAGGTGCGTC GCTCATGTCCTCCCTTTCCAA | NM_001247102 |
| *SlGST* | TGTGGACAACAAAGGGCGAA CCTCTGTGCTGAAGTTACCGT | LOC101244597 |
| *SlPIN1* | TCAAGAGGGCCAACTCCAAG  CTCTTGTGTGCTTTGTTGCCA | NM_001247234 |
| *SlPIN2* | CCAACACCGCGAACTTCTAAT TCCTTTTTCCTTTGCCCTACA | NM_001247241 |
| *SlPIN3*  *SlPIN4*  *SlPIN5*  *SlPIN6*  *SlPIN7*  *SlPIN8*  *SlPIN9*  *SlPIN10*  *SlLAX1*  *SlLAX2*  *SlLAX3*  *SlLAX4*  *SlLAX5*  *SlJAV1*  *SlWRKY51*  *SlRBCS-3B* | CAGCAACAACAAAATGGTAAAGC TTCGTTGGCACTAAAATCCGT  AAAGAGGGACCCACTGGACT  TCATAACACTAGCCGGAGGC  CATGGCTTTGCGTGGGAAAA  GCTTGGGGTAATGCAGCTTG  CTCACTAGACGGCCGGAATC  ATGCCCTAGGTGTGATCCCT  GGAATTGGACATCACTCAAAAGA  AGTTTCAGTAGCAGTCCTGGGA  GGTGGGGGAAACTCTTTTCA  TTCCTTTGGGCTTCAGTTTG  TTGTTCATGGCATTGTCGCC  AGAGCTGCCTGAACGATAGC  TCAAAGGCAGGAAGTGGCAT  GGGGTAATGCTGCCTGAAGT  AACTGTAGCGGCTCTGGTTC  ACGCCCAGTAAACTGCTGAA  TACTGTCGCATCGCTCCTTC  GATGCTGCTGATGGAAGGGT  ATGCTTTGGCTTTGTTGCCA  AGGGAGTCTTGCCATTGCTC  GCTTGGTTCAGCTGTGCTTC  GAGGTATGCAGTCCAGCTCC  GATGGGCTGCTAGGGAAACA  GTGGCACAACAAGCACCAAA  AGACGACGATCAAGGGCTTC  AGTAGCCCCCTTGTGATGGT  GTGGGGGATGCAATGTGAAGA  AGGTGAAGATTGTGAAGGGCA  AGCAACGGTGGAAGAGTCAG  ACGGTACACAAATCCGTGCT | NM_001368353  NM_001247255  NM_001247263  NM_001247270  NM_001247275  XM_010317549  NM_001247291  NM_001247301  NM_001246990  NM_001247746  NM_001247753  NM_001247759  NM_001246995  LOC104648424  LOC101258361  NM_001309210 |
