# Supplementary material for: SlJAZ10 and SlJAZ11 mediate dark-induced leaf senescence and regeneration
Source: PLoS Genet. 2022 Jul 13;18(7):e1010285. doi: 10.1371/journal.pgen.1010285 (PMC9278786; doi:10.1371/journal.pgen.1010285)
Supplement: S4 Table — (DOCX) [file pgen.1010285.s004.docx]

**S4 Table**. Primers used for construction of Y2H vectors.

| Genes | Forward primer (5' to 3', top),  reverse primer (5' to 3', bottom) | Accession |
| --- | --- | --- |
| *SlJAZ10* | GGAATTCCATATGATGAGAAGAAAGTGTAATTTGGAAC  CGGGATCCCTAGTGATGATATGGAGAAGTTATTTG | LOC101252609 |
| *SlJAZ11* | CGGAATTCATGAGAAGAAATTGTAATTTGGAGT  CGGGATCCCTAGTGATGATATGGCGAAGTTG | LOC101253212 |
| *SlJAV1* | CGGAATTCATGAGTGAAACCATGCCAAATAAC  CGGGATCCTTAGAAGGTGAAATTATTTTCCGATT | LOC104648424 |
| *SlWRKY51*  *SlRBCS-3B* | CGGAATTCATGGAAAATTTTCCCTATAGCTCATC  CGGGATCCCTAAAGGTGAAGATTGTGAAGGGC  CGGAATTCATGGCTTCCTCTATAGTTTCTTCAG  CGGGATCCGTATCCTTCGGGCTTGTAAGC | LOC101258361  NM_001309210 |

Primers used for construction of BiFC vectors.

| Genes | Forward primer (5' to 3', top),  reverse primer (5' to 3', bottom) | Accession |
| --- | --- | --- |
| *SlJAZ10* | CGGGATCCATGAGAAGAAAGTGTAATTTGGAAC  GCTCTAGAGTGATGATATGGAGAAGTTATTTG | LOC101252609 |
| *SlJAZ11* | CGGGATCCATGAGAAGAAATTGTAATTTGGAGT  GCTCTAGAGTGATGATATGGCGAAGTTG | LOC101253212 |
| *SlJAV1* | CGGGATCCATGAGTGAAACCATGCCAAATAA  GCTCTAGAGAAGGTGAAATTATTTTCCGATTT | LOC104648424 |
| *SlWRKY51*  *SlRBCS-3B* | CGGGATCCATGGAAAATTTTCCCTATAGCTCATC  GCTCTAGAAAGGTGAAGATTGTGAAGGGC  CGGGATCCATGGCTTCCTCTATAGTTTCTTCAG GCTCTAGAGTATCCTTCGGGCTTGTAAGC | LOC101258361  NM_001309210 |

Primers used for construction of Dual-luciferase assay.

| Genes | Forward primer (5' to 3', top),  reverse primer (5' to 3', bottom) | Accession |
| --- | --- | --- |
| *SlJAZ10* | GCTCTAGAATGAGAAGAAAGTGTAATTTGGAAC CCCAAGCTTCTAGTGATGATATGGAGAAGTTATTTG | LOC101252609 |
| *SlJAZ11* | GCTCTAGAATGAGAAGAAATTGTAATTTGGAGT  CCCAAGCTTCTAGTGATGATATGGCGAAGTTG | LOC101253212 |
| *SlJAV1* | GCTCTAGAATGAGTGAAACCATGCCAAATAAC  CCCAAGCTTTTAGAAGGTGAAATTATTTTCCGATT | LOC104648424 |
| *SlWRKY51*  *SlRBCS-3B*  *SlAOC*  *SlAOS* | GCTCTAGAATGGAAAATTTTCCCTATAGCTCATC  CCCAAGCTTCTAAAGGTGAAGATTGTGAAGGGC  GCTCTAGAATGGCTTCCTCTATAGTTTCTTCAG  CCCAAGCTTTTAGTATCCTTCGGGCTTGTA  GGGGTACCTAGACTCCAAAGCAGGTAACGGT  CCCAAGCTTGTTGGTTCTAGCGGTCAAAAAAG  GGGGTACCATAAATGAAAAGAGGGGGGAATG  CCCAAGCTTTCAAATCATTATCCGTTTTCCGT | LOC101258361  NM_001309210  NM_001247090  NM_001247904 |
