# Supplementary material for: SlJAZ10 and SlJAZ11 mediate dark-induced leaf senescence and regeneration
Source: PLoS Genet. 2022 Jul 13;18(7):e1010285. doi: 10.1371/journal.pgen.1010285 (PMC9278786; doi:10.1371/journal.pgen.1010285)
Supplement: S5 Table — (DOCX) [file pgen.1010285.s005.docx]

**S5 Table**. Primers used for construction of Pull-down assay.

| Genes | Forward primer (5' to 3', top),  reverse primer (5' to 3', bottom) | Accession |
| --- | --- | --- |
| *SlJAZ10* | CGGGATCCATGAGAAGAAAGTGTAATTTGGAACTCA  CCGCTCGAGTGATGATATGGAGAAGTTATTTGAA | LOC101252609 |
| *SlJAZ11* | CGGGATCCATGAGAAGAAATTGTAATTTGGAGTTTAG  CCGCTCGAGTGATGATATGGCGAAGTTGTTTGA | LOC101253212 |
| *SlRBCS-3B* | CGGGATCCATGGCTTCCTCTATAGTTTCTTCAGC  CCGCTCGAGTTAGTATCCTTCGGGCTTGTAA | NM_001309210 |
