# Supplementary material for: SlJAZ10 and SlJAZ11 mediate dark-induced leaf senescence and regeneration
Source: PLoS Genet. 2022 Jul 13;18(7):e1010285. doi: 10.1371/journal.pgen.1010285 (PMC9278786; doi:10.1371/journal.pgen.1010285)
Supplement: S6 Table — (DOCX) [file pgen.1010285.s006.docx]

**S6 Table**. Primers used for construction of Y1H.

| Genes | Forward primer (5' to 3', top),  reverse primer (5' to 3', bottom) | Accession |
| --- | --- | --- |
| *SlJAZ10* | GGAATTCCATATGATGAGAAGAAAGTGTAATTTGGAAC  CGGGATCCCTAGTGATGATATGGAGAAGTTATTTG | LOC101252609 |
| *SlJAZ11* | CGGAATTCATGAGAAGAAATTGTAATTTGGAGT  CGGGATCCCTAGTGATGATATGGCGAAGTTG | LOC101253212 |
| *SlJAV1* | CGGAATTCATGAGTGAAACCATGCCAAATAAC  CGGGATCCTTAGAAGGTGAAATTATTTTCCGATT | LOC104648424 |
| *SlWRKY51*  *SlRBCS-3B*  *SlAOC*  *SlAOS* | CGGAATTCATGGAAAATTTTCCCTATAGCTCATC  CGGGATCCCTAAAGGTGAAGATTGTGAAGGGC  CGGAATTCATGGCTTCCTCTATAGTTTCTTCAG  CGGGATCCGTATCCTTCGGGCTTGTAAGC ACGCGTCGACTAGACTCCAAAGCAGGTAACGGT  GGGGTACCGTTGGTTCTAGCGGTCAAAAAAG  ACGCGTCGACATAAATGAAAAGAGGGGGGAATG  GGGGTACCTCAAATCATTATCCGTTTTCCGT | LOC101258361  NM_001309210  NM_001247090  NM_001247904 |
