# Supplementary figures and images for: SlJAZ10 and SlJAZ11 mediate dark-induced leaf senescence and regeneration
Source: PLoS Genet. 2022 Jul 13;18(7):e1010285. doi: 10.1371/journal.pgen.1010285 (PMC9278786; doi:10.1371/journal.pgen.1010285)

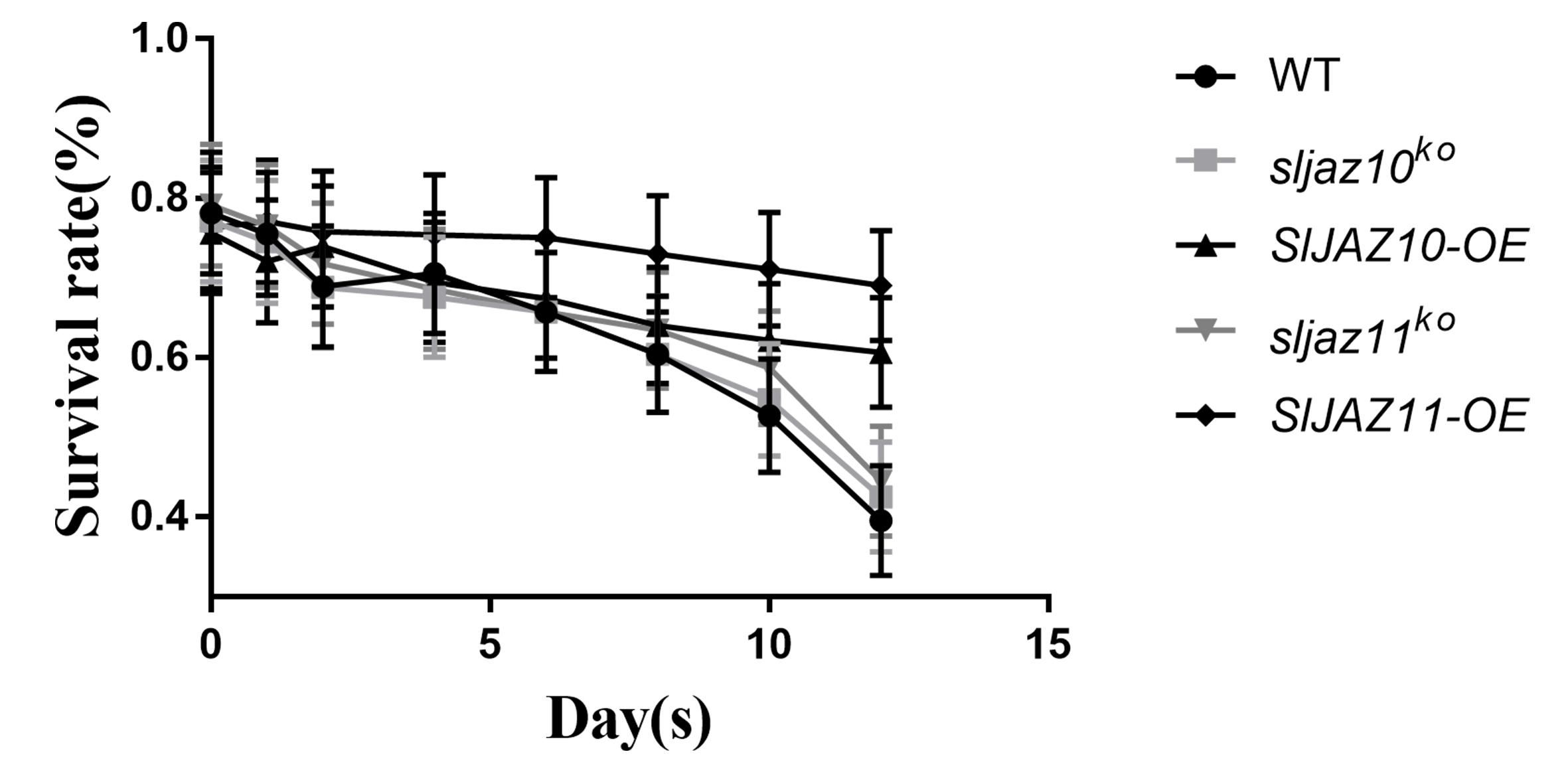

Supplement: S1 Fig — (TIF) [file pgen.1010285.s008.tif]

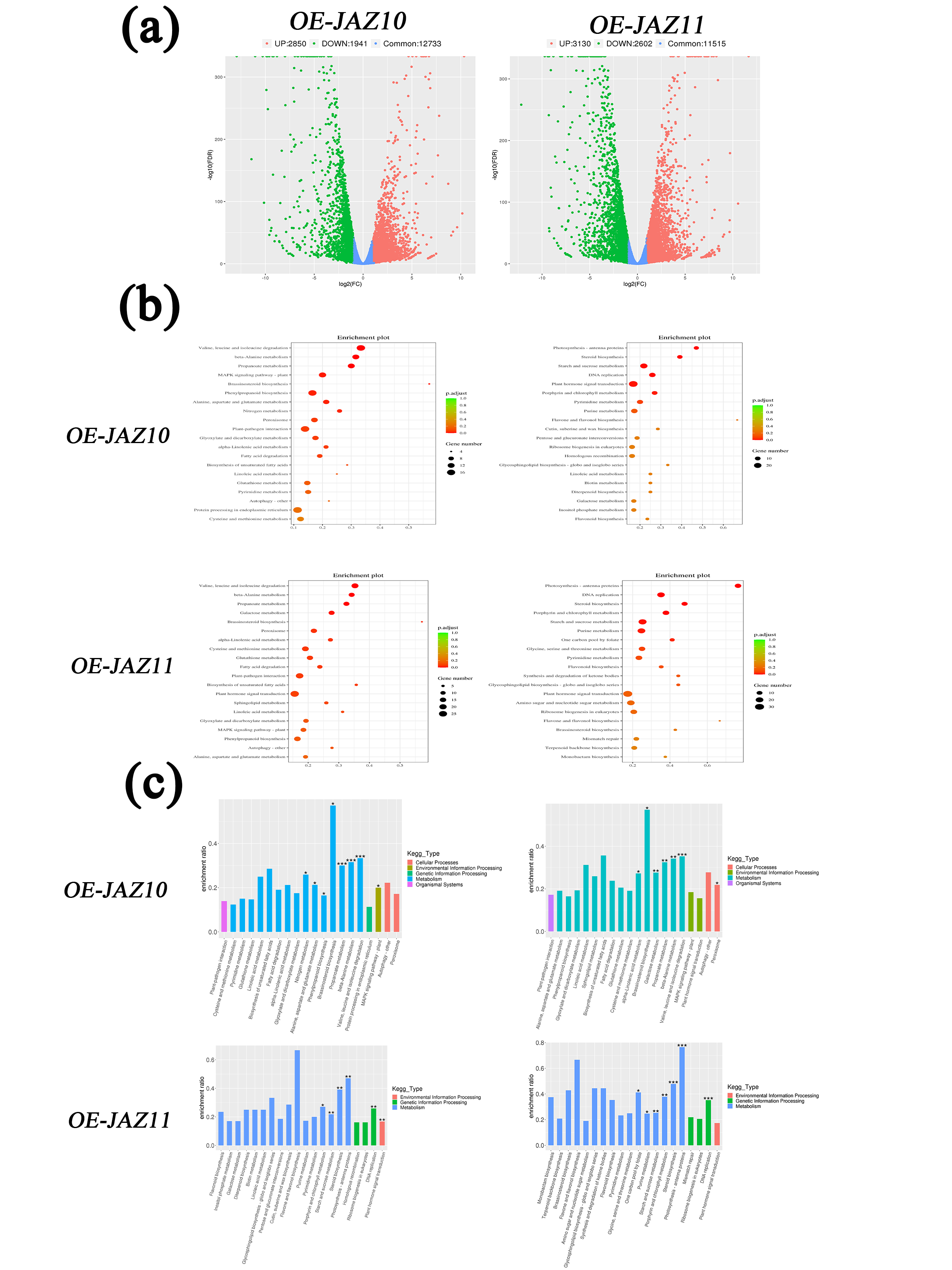

Supplement: S2 Fig — (a) Volcano plot for differential gene expression. (b) GO enrichment analysis. (c) KEGG and KEGG enrichment analysis of the DEPs. (TIF) [file pgen.1010285.s009.tif]

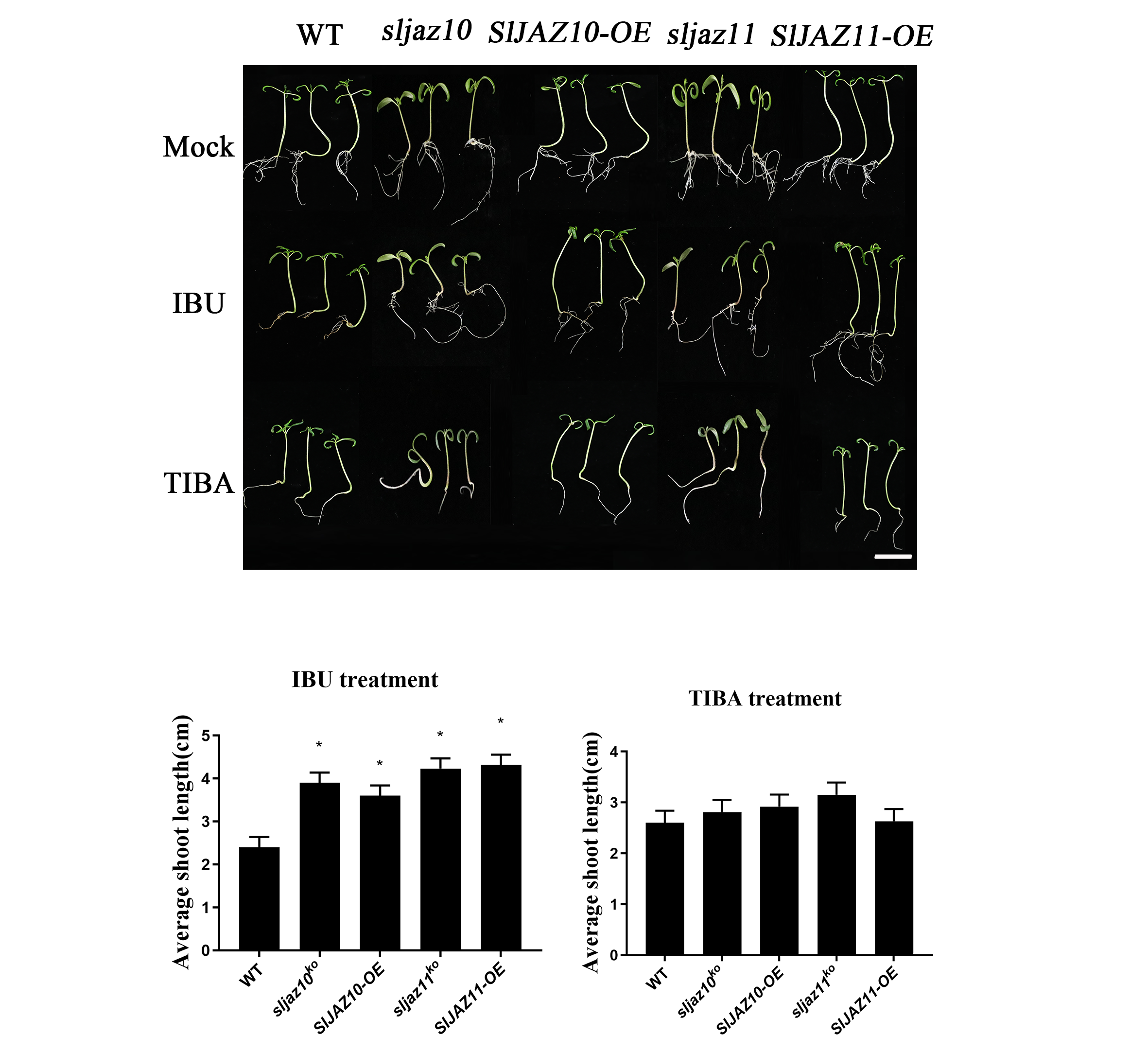

Supplement: S3 Fig — (TIF) [file pgen.1010285.s010.tif]

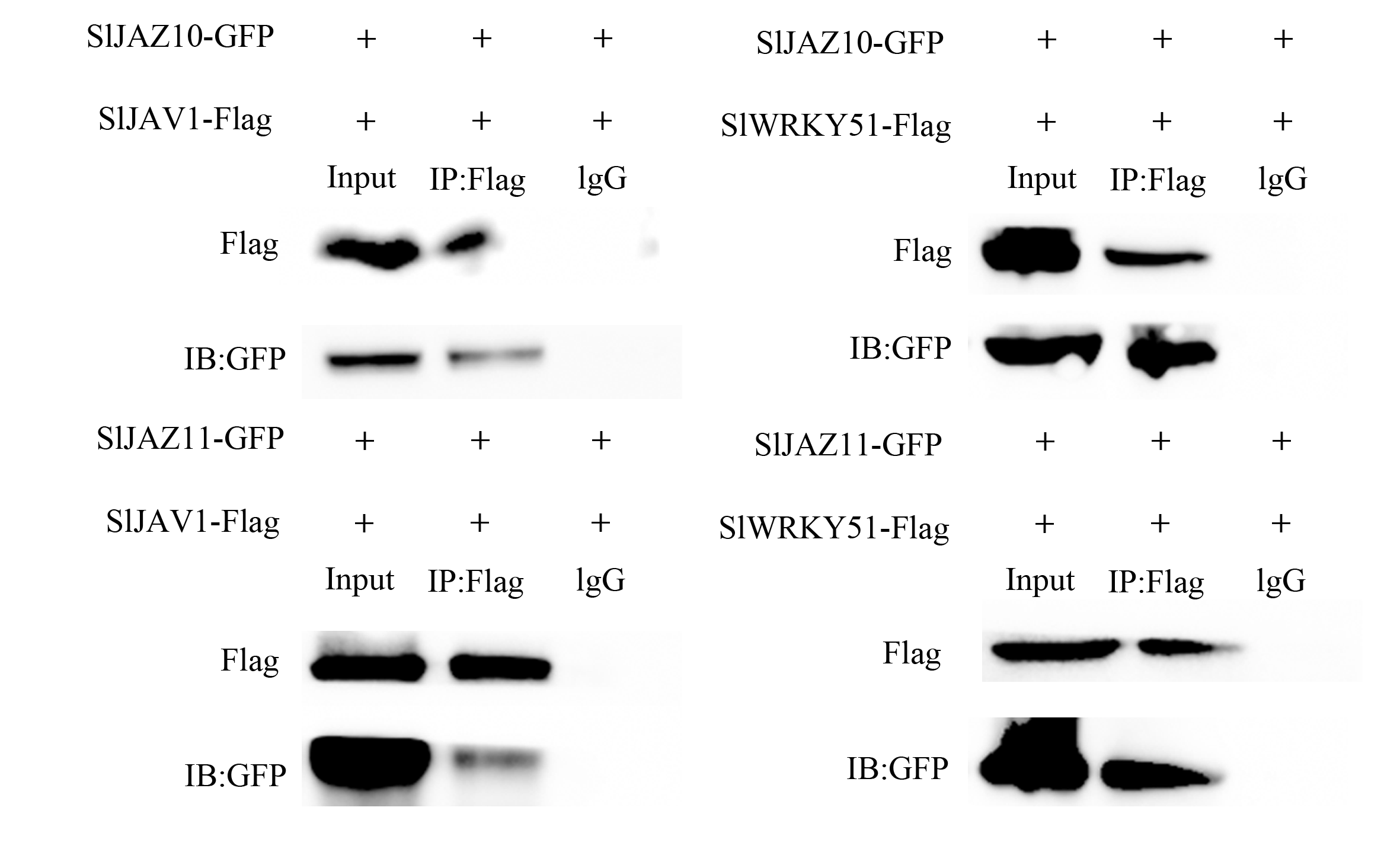

Supplement: S4 Fig — (TIF) [file pgen.1010285.s011.tif]

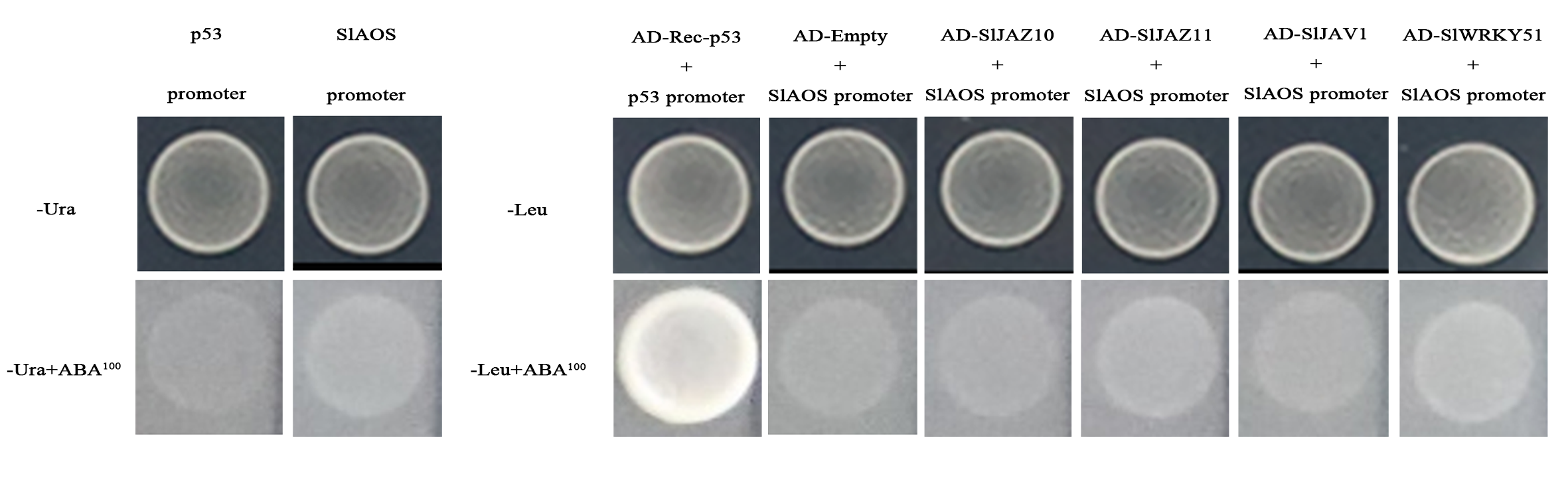

Supplement: S5 Fig — (TIF) [file pgen.1010285.s012.tif]

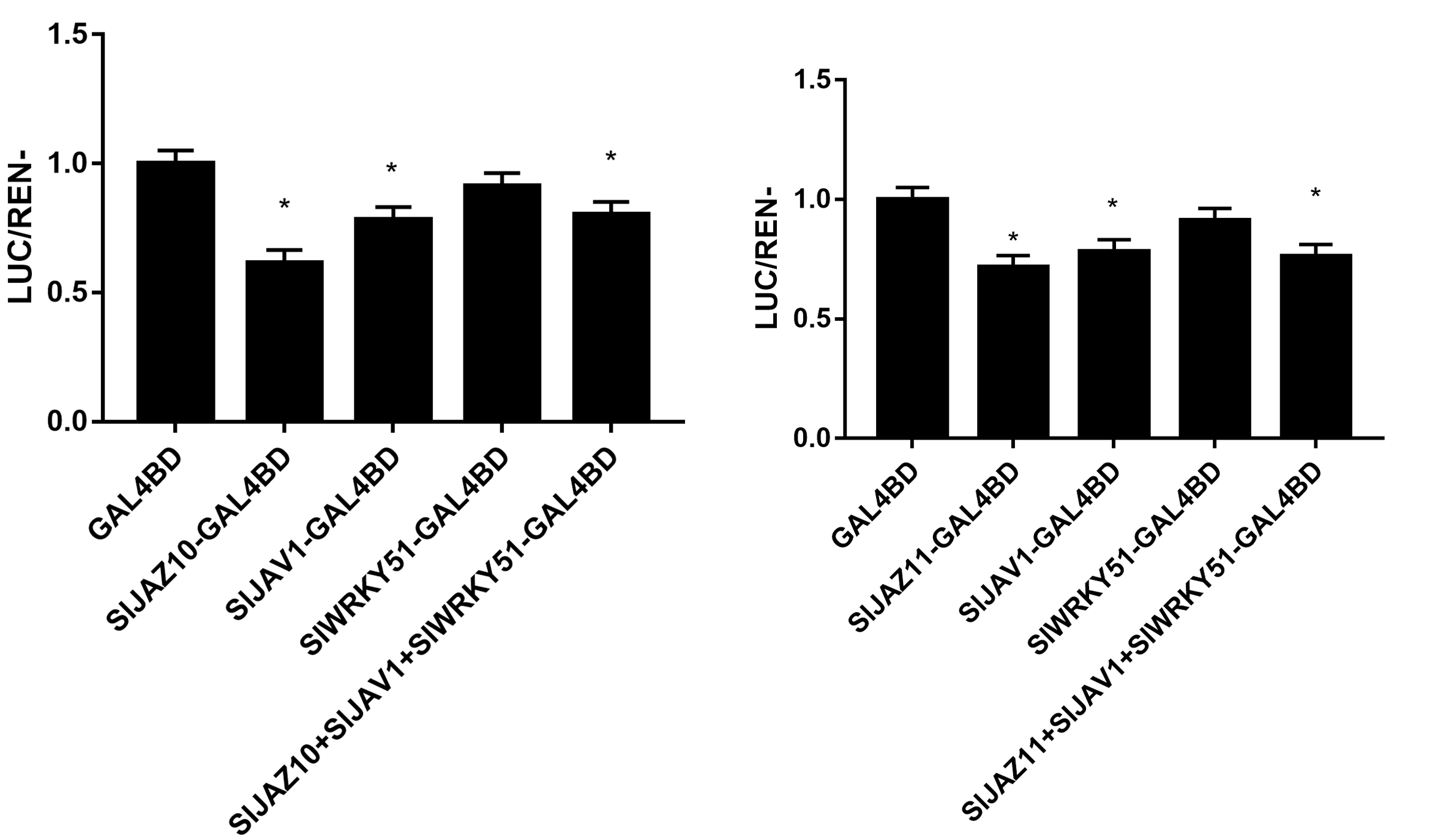

Supplement: S6 Fig — (TIF) [file pgen.1010285.s013.tif]

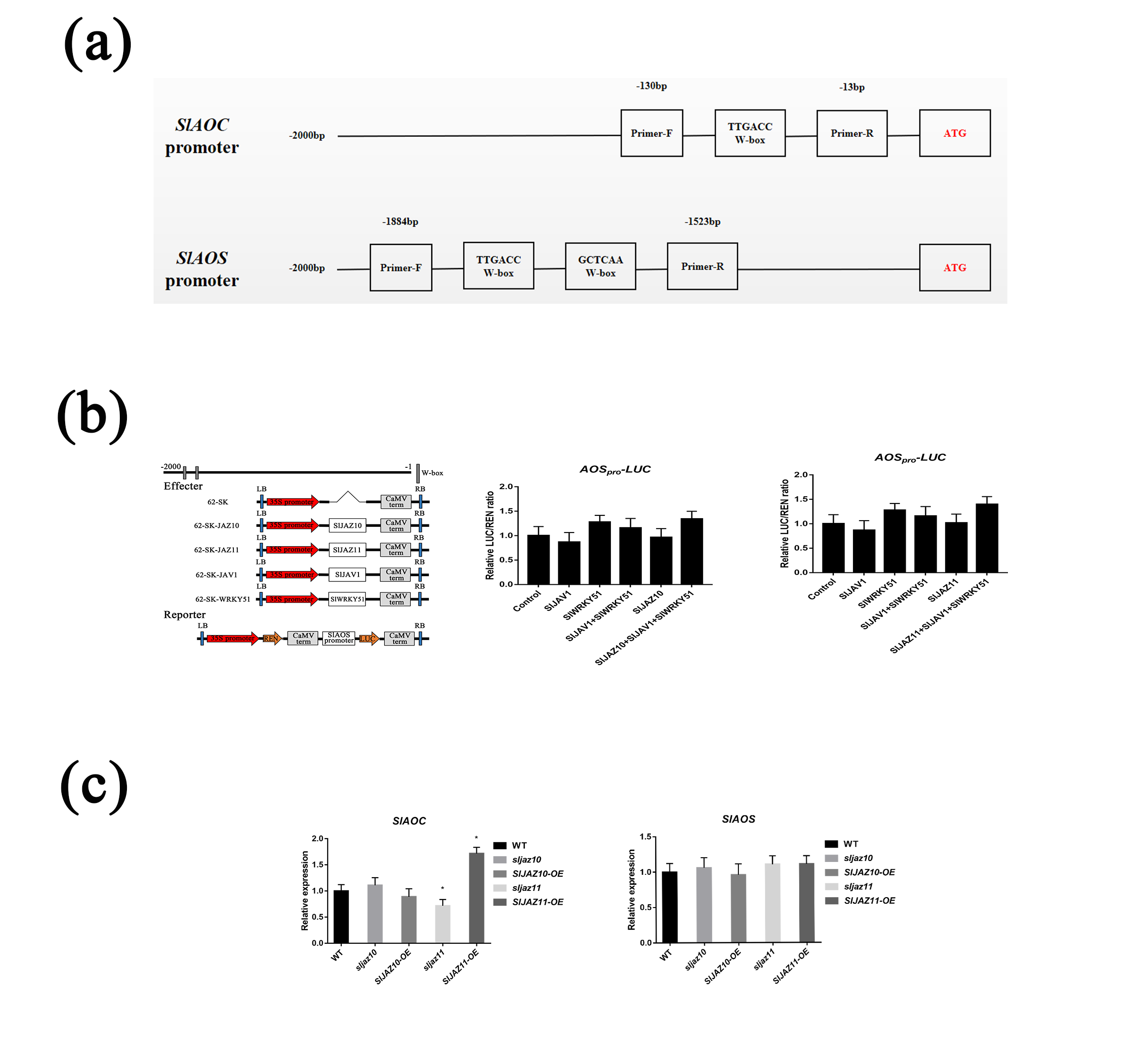

Supplement: S7 Fig — (a) Binds the SlAOC and SlAOS promoter region. (b) SlJAZ10-SlJAV1-SlWRKY51 and SlJAZ11-SlJAV1-SlWRKY51complex effectively suppresses the expression of SlAOSPro-LUC in N. benthamiana transient expression assay. (c) Relative gene expression of SlAOC and SlAOS in WT, sljaz10, SlJAZ10-OE, sljaz11 and SlJAZ11-OE. (TIF) [file pgen.1010285.s014.tif]

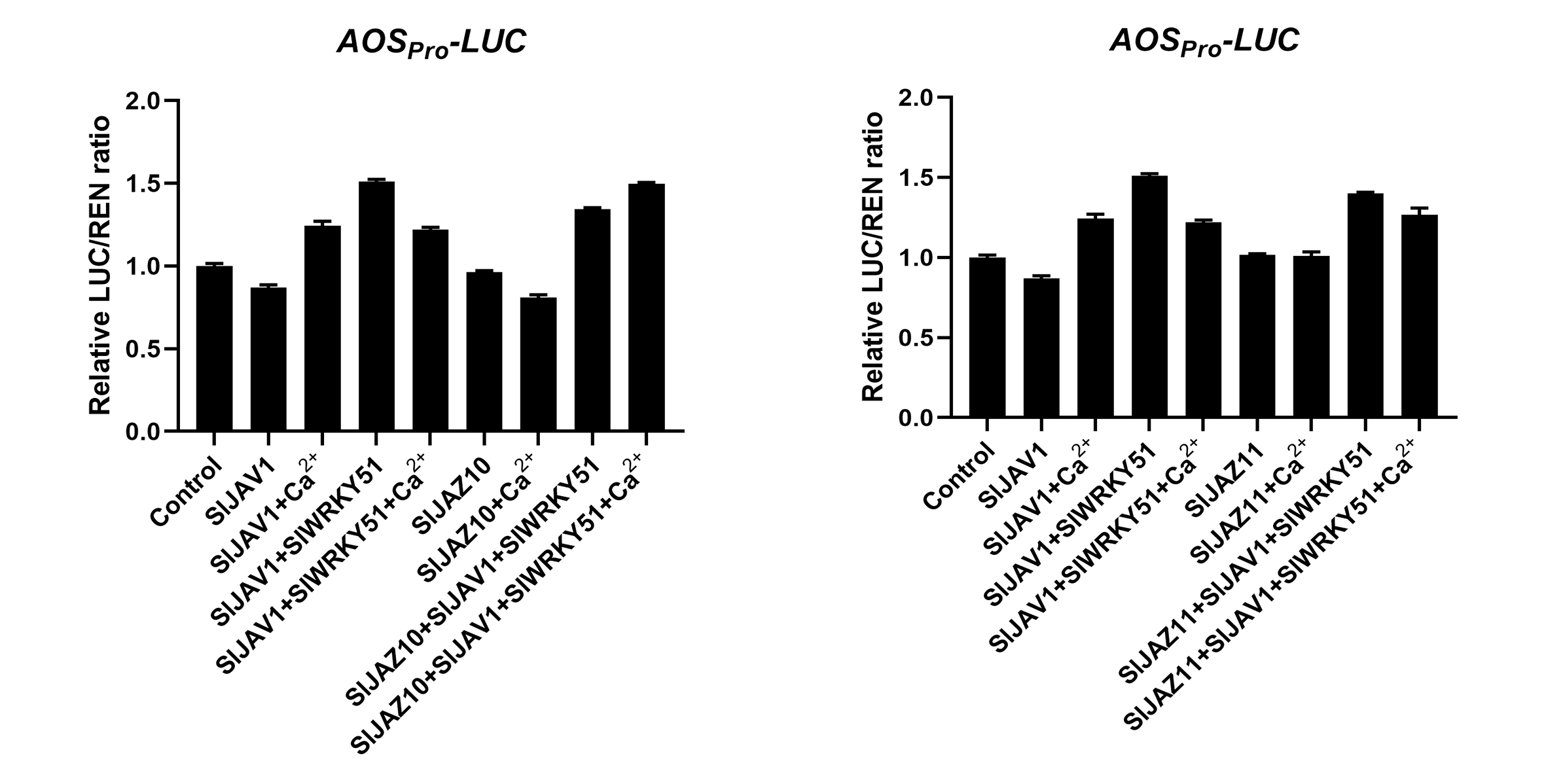

Supplement: S8 Fig — (TIF) [file pgen.1010285.s015.tif]

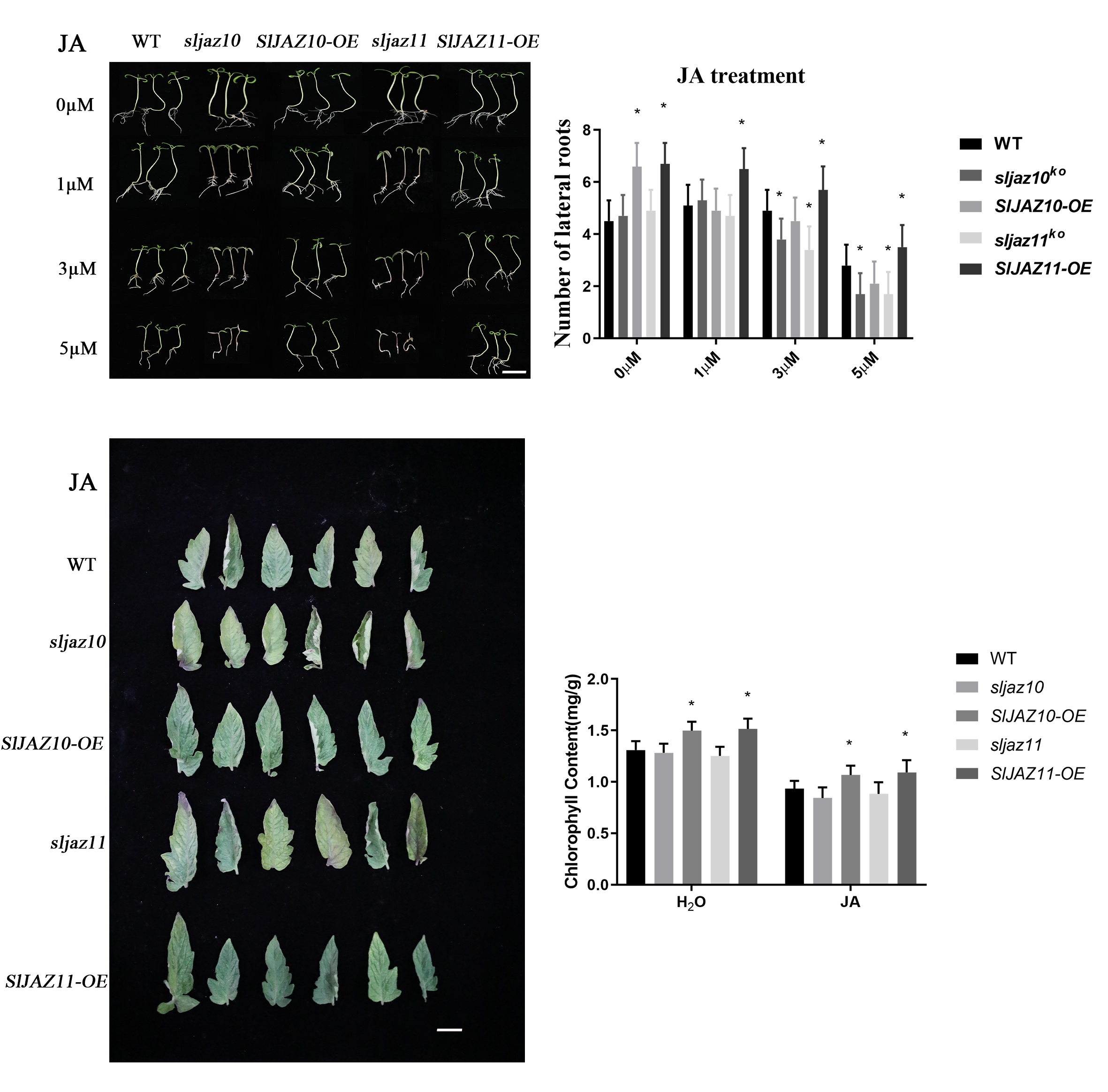

Supplement: S9 Fig — (TIF) [file pgen.1010285.s016.tif]

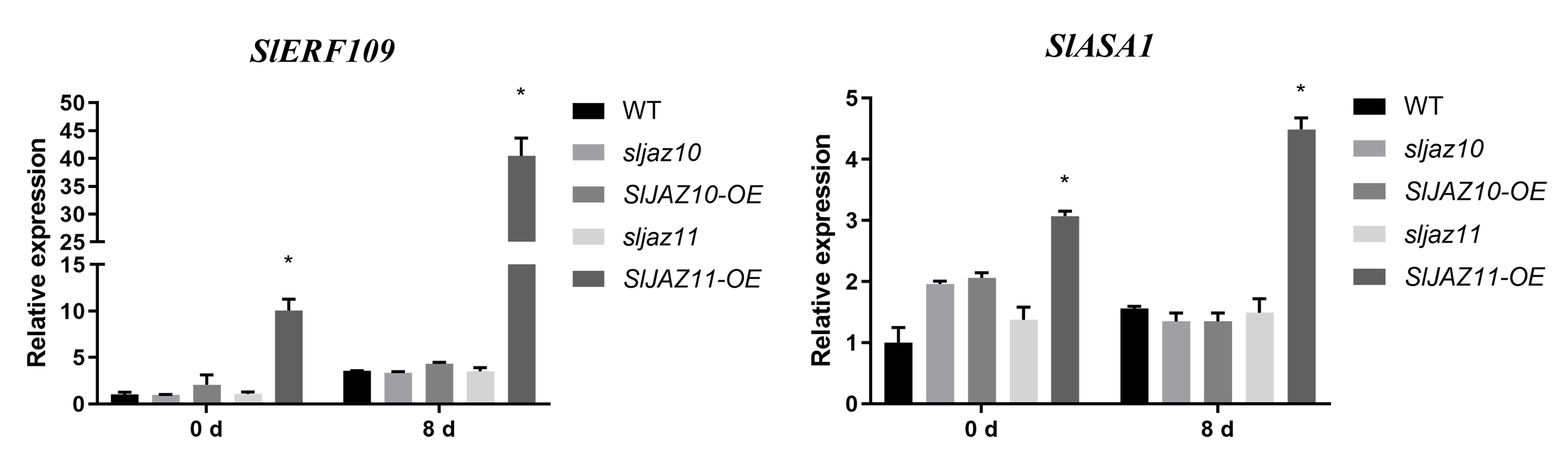

Supplement: S10 Fig — (TIF) [file pgen.1010285.s017.tif]
